# Early‐life antibiotic exposure aggravates hepatic steatosis through enhanced endotoxemia and lipotoxic effects driven by gut *Parabacteroides*

**DOI:** 10.1002/mco2.70104

**Published:** 2025-02-17

**Authors:** Xi Zhang, Darren Chak Lun Chan, Jie Zhu, Daniel Zhen Ye Sin, Ye Peng, Matthew Kwok Leong Wong, Wenyi Zhu, Yee Tsui, Andrea M. Haqq, Joseph Y. Ting, Anita Kozyrskyj, Francis Ka Leung Chan, Siew Chien Ng, Hein Min Tun

**Affiliations:** ^1^ Microbiota I‐Center (MagIC) Hong Kong SAR China; ^2^ Jockey Club School of Public Health and Primary Care Faculty of Medicine, The Chinese University of Hong Kong Hong Kong SAR China; ^3^ Li Ka Shing Institute of Health Sciences Faculty of Medicine, The Chinese University of Hong Kong Hong Kong SAR China; ^4^ HKU‐Pasteur Research Pole, School of Public Health LKS Faculty of Medicine, The University of Hong Kong Hong Kong SAR China; ^5^ Department of Pediatrics University of Alberta Edmonton Canada; ^6^ Centre for Gut Microbiota Research The Chinese University of Hong Kong Hong Kong SAR China; ^7^ Department of Medicine and Therapeutics Faculty of Medicine The Chinese University of Hong Kong Hong Kong SAR China

**Keywords:** antibiotics, early life, gut microbiota, lipotoxicity, metabolic dysfunction‐associated steatotic liver disease

## Abstract

Compelling evidence supports a link between early‐life gut microbiota and the metabolic outcomes in later life. Using an early‐life antibiotic exposure model in BALB/c mice, we investigated the life‐course impact of prenatal and/or postnatal antibiotic exposures on the gut microbiome of offspring and the development of metabolic dysfunction‐associated steatotic liver disease (MASLD). Compared to prenatal antibiotic exposure alone, postnatal antibiotic exposure more profoundly affected gut microbiota development and succession, which led to aggravated endotoxemia and metabolic dysfunctions. This was primarily resulted from the overblooming of gut *Parabacteroides* and hepatic accumulation of cytotoxic lysophosphatidyl cholines (LPCs), which acted in conjunction with LPS derived from *Parabacteroides distasonis* (LPS_PA) to induce cholesterol metabolic dysregulations, endoplasmic reticulum (ER) stress and apoptosis. Integrated serum metabolomics, hepatic lipidomics and transcriptomics revealed enhanced glycerophospholipid hydrolysis and LPC production in association with the upregulation of PLA2G10, the gene controlling the expression of the group X secretory Phospholipase A2s (sPLA2‐X). Taken together, our results show microbial modulations on the systemic MASLD pathogenesis and hepatocellular lipotoxicity pathways following early‐life antibiotic exposure, hence help inform refined clinical practices to avoid any prolonged maternal antibiotic administration in early life and potential gut microbiota‐targeted intervention strategies.

## Graphical abstract

Early‐life antibiotic exposure caused the overblooming of gut *Parabacteroides* and hepatic accumulation of cytotoxic lysophosphatidyl cholines (LPCs), which acted in conjunction with LPS derived from *Parabacteroides distasonis* (LPS_PA) to induce cholesterol accumulation, endoplasmic reticulum stress and apoptosis through ATF6 pathway. Excessive LPS_PA increased the group X secretory Phospholipase A2s, which is associated with enhanced glycerophospholipid hydrolysis and LPC production. Icons were made using BioRender.

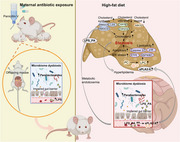

## 1 INTRODUCTION

The early‐life gut microbiome plays an important role in the healthy development of the immune system and metabolism of the host. Indeed, early‐life gut dysbiosis affects the risks of developing multiple chronic diseases in later life including obesity and liver steatosis.^1^, ^2^ Several prenatal and postnatal factors affect gut microbiota succession and development in neonates such as delivery mode, neonatal diets (e.g., breastfeeding and solid food intake), and antibiotic exposure,^3^, ^4^, ^5^ the latter of which is an increasing concern. An estimated 20–30% of pregnant women and their newborns worldwide receive antibiotics to prevent severe neonatal bacterial infections and/or postpartum infections following cesarean delivery; most infants receive antibiotics in their first year of life.^6^, ^7^ Worryingly, gut microbiota dysbiosis in early life may lead to the impaired development of mucosal and systemic immunities and increase the risks of suffering from immune‐mediated diseases and metabolic outcomes in later life, including metabolic dysfunction‐associated steatotic liver disease (MASLD).^6^, ^8^, ^9^, ^10^, ^11^

MASLD, formerly known as nonalcoholic fatty liver disease, is a metabolic disorder where hepatic steatosis is accompanied by overweight/obesity, type 2 diabetes mellitus, or other metabolic disorders.^12^ The global prevalence of MASLD is currently estimated to be 39.22%,^13^ and around 20% of patients rapidly and asymptomatically progress to advanced fibrosis,^14^ a leading cause for hepatocellular carcinoma and cardiovascular complications.^15^ Sufferers with chronic liver disease are closely associated with the development of severe COVID­19.^16^ Of concern, there is no specific pharmacological therapy currently available. The etiology of MASLD consists of complex interactions between environmental factors, genetics, and metabolic risk factors including obesity and microbiome dysbiosis.^17^, ^18^, ^19^ Microbiome dysbiosis has been linked to dysregulated lipid metabolism^20^, ^21^, ^22^, ^23^, ^24^; abnormal lipid and cholesterol metabolism then drives steatosis progression through lipotoxic effects [e.g., the induction of endoplasmic reticulum (ER) stress] that cause hepatocellular damage and ultimately cell death.^25^, ^26^ Direct antibiotic exposure in early life has been shown to enhance hepatic metabolic disorders, adiposity, and immunity defects through gut microbiome dysbiosis.^27^, ^28^, ^29^ However, the respective contributions of prenatal and/or postnatal antibiotic exposure on the development of metabolic syndrome in offspring have not yet been clarified. While several human studies linked early‐life antibiotic exposure to childhood obesity risk and infant microbiota alterations,^30^, ^31^, ^32^ the life‐long effect of maternal antibiotic exposure remains unknown, probably due to the long time‐span and vague information of the duration and the type of antibiotic usage.

Given the lack of suitable clinical evidence, we therefore implemented a BALB/c mouse model to comparatively characterized both short‐ and long‐term impacts of prenatal and postnatal maternal antibiotic exposure on the gut microbiome of offspring. Penicillin V was administered to female mice, as this β‐lactam antibiotic is one of the most frequently prescribed antibiotics worldwide to prevent severe bacterial infections during pregnancy or following cesarean delivery.^33^, ^34^ The hepatocellular pathogenic responses were investigated through a multiomics analysis and associated with the cytotoxic lipids lysophosphatidyl cholines (LPCs) and microbiota perturbations generated by early‐life antibiotic exposure. We then evaluated the enrichment of *Parabacteroides* on gut barrier function, the induction of endotoxemia, and the subsequent metabolic dysfunctions and hepatic cell death effect.

## 2 RESULTS

### 2.1 Early‐life antibiotic exposure disrupted the intergenerational succession of gut microbiota from dams to offspring

**FIGURE 1:**
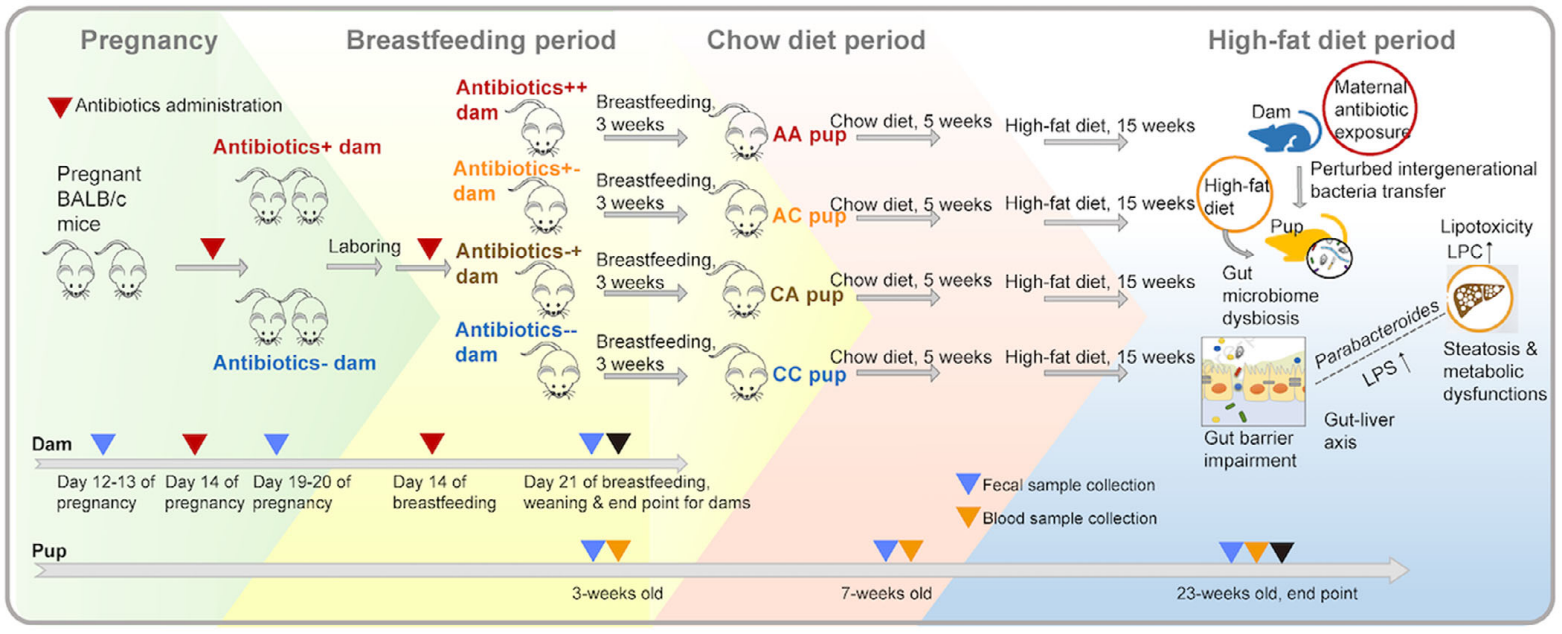
Schematic overview of study design. Assigned female BALB/c mice were exposed to Penicillin V via drinking water at 0.25 mg/mL in their late pregnancy (the last week prior to laboring), late lactation period (the last week prior to weaning), or during both the pregnancy and breastfeeding period. Pups were weaned at Day 21 and retained males were then assigned into 4 groups according to their early‐life antibiotic exposure status. All groups of pups underwent a 10%  kcal fat standard chow diet period for 5 weeks, then shifted to a 45%  kcal fat high‐fat diet until the end of the experiment. Fecal samples were collected from each dam before and after antibiotic administration during pregnancy and the lactation period, and from each pup during the breastfeeding, chow diet, and high‐fat diet periods for gut microbiota profiling analysis. All the mice were sacrificed at the endpoint for tissue collection. LPS, lipopolysaccharides; LPC, lysophosphatidyl choline.

**FIGURE 2:**
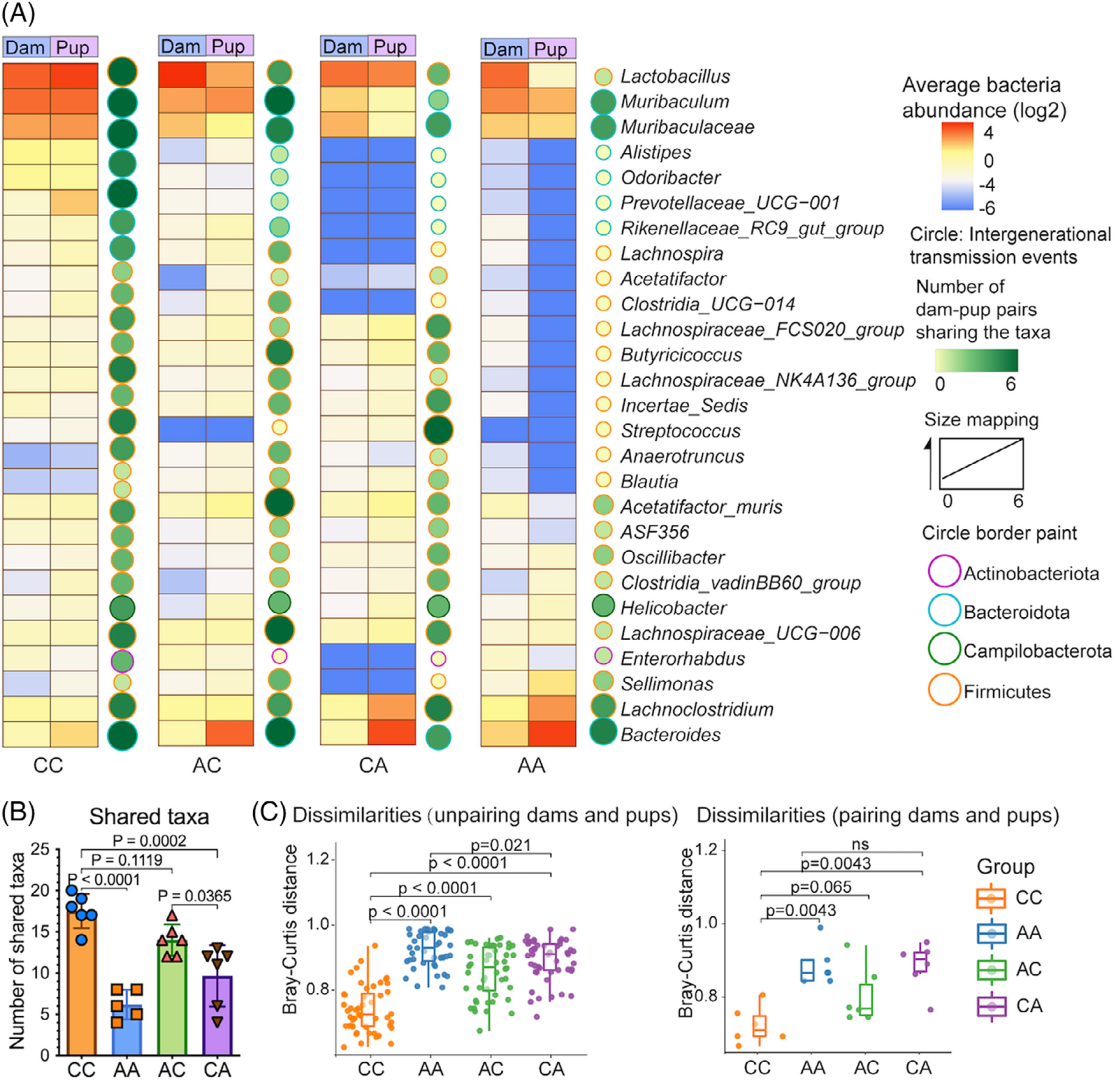
Intergenerational microbiota transfer fidelities varied in mice with prenatal and/or postnatal antibiotic exposure. (A) Intergenerational distribution and expression of each bacteria taxa. Each pup and dam were paired up to identify the number of shared bacterial taxa and their abundances. The heatmap squares were generated based on the average abundance of taxa from 16S rRNA microbiome profiling. The size of the circle is proportional to the number of dam‐pup pairs with intergenerational transfer of the respective taxa. (B) The number of shared taxa among paired dams and pups. (C) Bray–Curtis distance calculated by grouping all dams and pups under each treatment together (left panel) and pairing each dam and pup for matched comparison (right panel). Data point collected during breastfeeding (when pups reached 3 weeks) were used for analysis. The number of dam‐pup dyads for CC, AA, AC, CA group was 6, 5, 6, 6, respectively. *p* value was calculated by a Wilcoxon's rank‐sum test. AA, pups born by dams with prenatal and postnatal antibiotic exposure; AC, pups born by dams with only prenatal antibiotic exposure; CA, pups born by dams with only postnatal antibiotic exposure; CC, pups born by dams without antibiotic exposure.

We longitudinally monitored the gut microbiota profiles of mice exposed to antibiotics through maternal antibiotic administration prenatally and/or postnatally (Figure 1). To determine whether maternal antibiotic administration affected the intergenerational transfer of gut microbiota from dam to pup, we analyzed the shared bacterial taxa between the baseline pregnancy microbiota of dams and their respective offspring's microbiota during the breastfeeding period. Consistent with previous studies,^35^ the similarities of gut microbiota profiles varied among dam‐pup dyads in different antibiotic exposure groups (Figure 2, Table S1). The gut microbiota of the control group of pups born by dams without maternal antibiotic exposure (CC) demonstrated the highest transfer fidelity, as represented by a larger number of shared taxa and the occurrence of intergenerational transfer events among all dam‐pup dyads (Figure 2A, Table S1). While the transfer fidelity of the gut microbiota in the AC (pups born by dams with only prenatal antibiotic exposure) groups were at a comparable level to that of the CC group, those of the CA (pups born by dams with only postnatal antibiotic exposure) and AA (pups born by dams with both prenatal and postnatal antibiotic exposure) groups were largely dampened, as indicated by a smaller number of shared taxa between generations (Figure 2B, Table S1) and significantly higher dissimilarities in the overall gut microbiota composition (Figure 2C). For the dam‐pup pairs of the CC and AC groups, the majority of the bacterial taxa were almost identical. However, almost half of the taxa originally present in dams, including *Lactobacillus*, *Muribaculum*, *Alistipes*, and *Odoribacter*, were either diminished or depleted in their respective offspring in the CA and AA groups (Figure 2A). The gut microbiota of the AA group, which was exposed to antibiotics for the longest duration (during both prenatal and postnatal periods), showed the least transfer fidelity.

### 2.2 Short‐ and long‐term perturbations in gut microbiota of offspring followed early‐life maternal antibiotic administration

**FIGURE 3:**
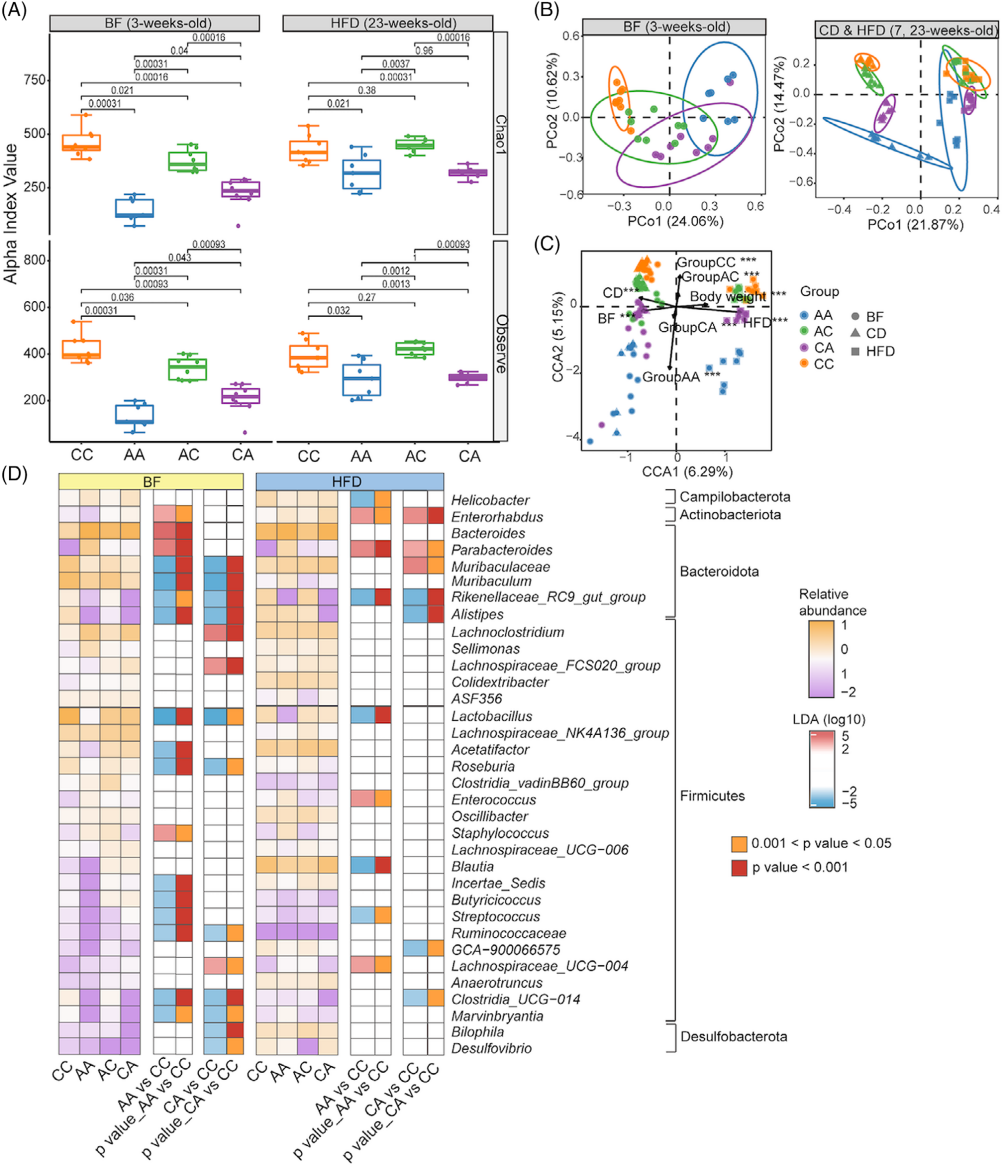
Prenatal and/or postnatal antibiotic exposure altered the gut microbiome composition and diversity in offspring mice. (A) α‐diversity of the gut flora of pups was calculated by Chao1 and Observe. (B, C) Principal coordinates analysis (PCoA) (B) and Canonical correlation analysis (C) showing the similarities in the gut microbiome community structures among pups. (D) Linear discriminant analysis effect size (LEfSe) showing the bacterial taxa altered by early‐life antibiotic exposure under dietary effects. The relative abundance of each taxon was graphed in the heatmap. We considered taxa with linear discriminant analysis (LDA) > 2 and *p* < 0.05 to be significant. The most significantly representative taxa were marked with blue (more abundant in CC) or red (more abundant in AA or CA). The number of pups within CC, AA, AC, CA group was 6, 5, 6, 6, respectively. BF samples were collected when pups were 3 weeks old; CD samples were collected when pups were 7 weeks old; HFD samples were collected when pups were 23 weeks old. BF, breastfeeding period; CD, chow diet period; HFD, high‐fat diet period.

We found both short‐ and long‐term effects of early‐life maternal antibiotic administration on the gut microbiota of offspring with different diets at both the neonatal and postweaning periods (Figure 3A‐D). Compared to the CC group, early‐life antibiotic exposure resulted in the depletion of microbiota species richness (Figure 3A). Moreover, the magnitude of microbiota depletion in the CA and AA groups was greater compared to that of AC group (Figure 3A). Both principal coordinates analysis (PCoA) and canonical correlation analysis (CCA) indicated that the gut microbiota composition of the AA group was similar to that of the CA group, while the gut microbiota composition of the AC group was more similar to that of the CC group (Figure 3C). *Firmicutes* and *Bacteroidetes* were the predominant bacterial phyla in all groups. To identify differential genera associated with postnatal antibiotic exposure, we used linear discriminant analysis effect size (LEfSE) to compare bacterial abundances at the genus level. The abundances of multiple bacterial genera such as *Rikenellaceae_RC9_gut_group*, *Alistipes*, and *Lactobacillus* were consistently diminished in the CA and AA groups under both breastfeeding and HFD. The abundances of *Enterorhabdus*, *Bacteroides*, *Parabacteroides*, *Lachnoclostridium*, *Lachnospiraceae_FCS020_group*, *Staphylococcus*, *Lachnospiraceae_UCG‐004*, *Muribaculaceae*, and *Enterococcus* were consistently enriched in the AA or CA groups [linear discriminant analysis (LDA) score > 2, *p* < 0.05] (Figure 3D).

By contrast, in dams, the effect of antibiotic administration was reversible: the altered gut microbiota returned to the baseline profile after the removal of antibiotic administration in different mouse groups (Figure S1).

### 2.3 Early‐life antibiotic exposure aggravated metabolic dysfunction through microbiome‐mediated mechanisms

**FIGURE 4:**
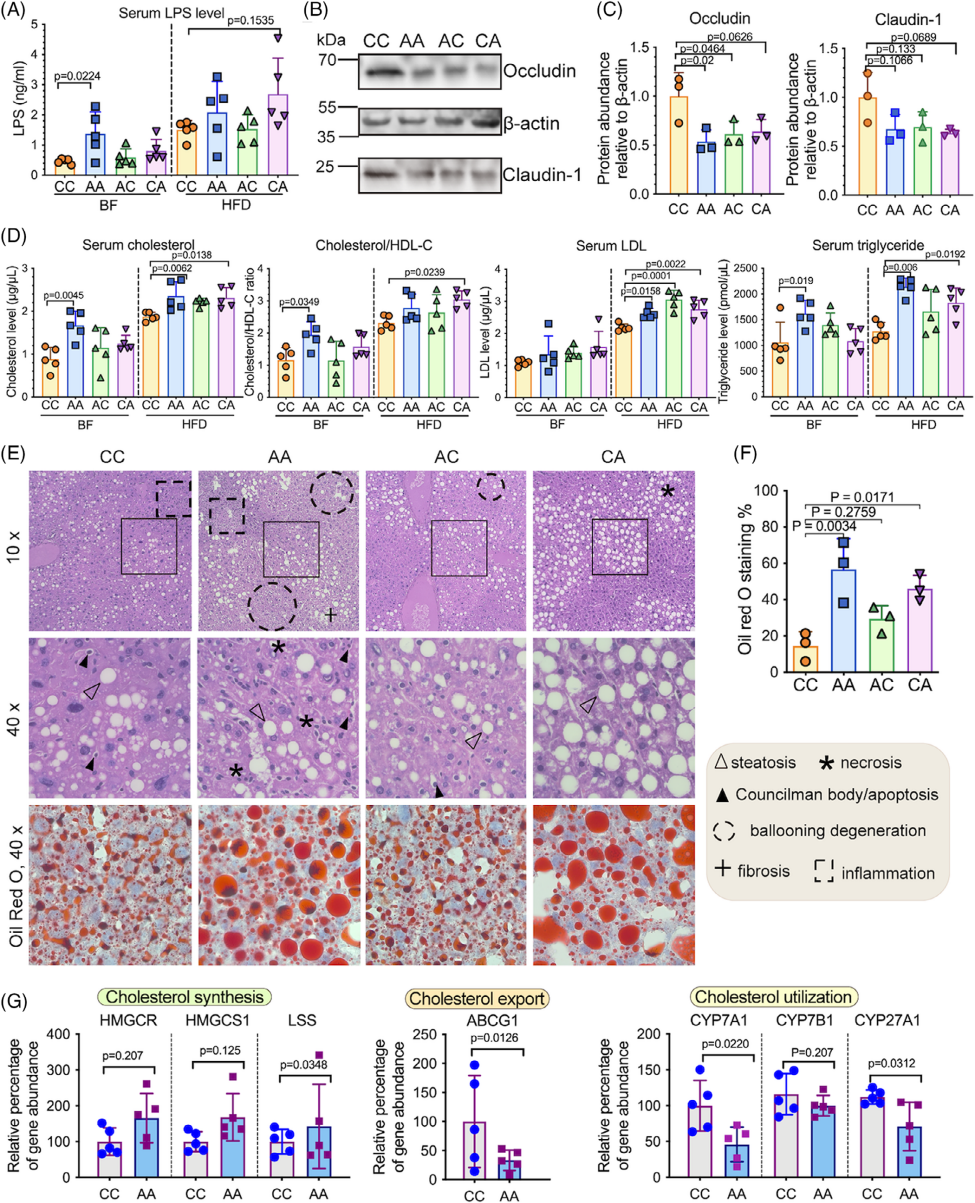
Postnatal antibiotic exposure aggravated the metabolic dysfunction in HFD‐fed mice. (A) Serum LPS level of pups before (during breastfeeding (BF)) and after HFD treatment. Data represented as mean ± SEM, *n* = 5, one‐way ANOVA. (B, C) Western blot image (B) and protein density quantification (C) of BALB/c pups’ ileal tight junction protein expression after HFD treatment. The abundance of occludin and claudin‐1 in each sample (C) was quantified relative to β‐actin in western blot images by ImageJ. Data represented as mean ± SEM, *n* = 3, one‐way ANOVA. (D) Serum total cholesterol, triglyceride, low‐density lipoprotein cholesterol (LDL‐C), and cholesterol/high‐density lipoprotein cholesterol (HDL‐C) ratios. Data represented as mean ± SEM, *n* = 5, one‐way ANOVA. (E) Representative H&E and ORO staining of the mice liver after HFD treatment. (F) Hepatic lipid deposition represented by the percentage of ORO positive‐stained area as quantified by ImageJ. Data represented as mean ± SEM, based on 3 images for each group, one‐way ANOVA. (G) The relative abundance of hepatic genes regulating cholesterol synthesis (HMGCR, HMGCS1, LSS), cholesterol export (ABCG1), and cholesterol utilization (CYP7A1, CYP7B1, CYP27A1) in CC and AA group after HFD treatment. Data represented as mean ± SEM, *n* = 5, Student’ s *t*‐test was used to determine significant differences for each gene. BF samples were collected when pups were 3 weeks old; HFD samples were collected when pups were 23 weeks old.

While HFD treatment did not significantly change body weight (Figure S2), we observed a trend of elevated serum lipopolysaccharides (LPS) in mice with early‐life antibiotic exposure, albeit not statistically significant (Figure 4A). Regardless, HFD treatment reduced levels of tight junction protein occludin in the ileum (Figure 4B and C), raised levels of serum cholesterol, triglyceride, low‐density lipoprotein cholesterol (LDL‐C), cholesterol/high‐density lipoprotein cholesterols (HDL‐C) ratio (Figure 4D) and hepatic lipid deposition (Figure 4E and F) in mice exposed to antibiotics in early life. Ileal barrier impairment, metabolic endotoxemia, and hepatic steatosis were more prominent in mice with postnatal antibiotic exposure (AA and CA mice) compared to mice with prenatal antibiotic exposure alone. Liver transcriptomics analysis indicated imbalanced lipid synthesis and utilization in antibiotics‐exposed mice, which may have contributed to steatosis development. Genes regulating cholesterol synthesis [3‐Hydroxy‐3‐Methylglutaryl‐CoA Reductase (HMGCR), 3‐Hydroxy‐3‐Methylglutaryl‐CoA Synthase 1 (HMGCS1), Lanosterol Synthase (LSS)] tended to increase in the livers of AA mice under HFD (Figure 4G). While the gene regulating cholesterol export and utilization, including ATP Binding Cassette Subfamily G Member 1 (ABCG1), and several rate‐limiting enzymes in cholesterol metabolism including Cholesterol 7α‐hydroxylase (CYP7A1), Cytochrome P450 Family 7 Subfamily B Member 1 (CYP7B1) and Cytochrome P450 Family 7 Subfamily A Member 1 (CYP27A1), were transcriptionally downregulated in AA group compared to mice without early‐life antibiotic exposure (CC) (Figure 4G). Interestingly, hepatic expression of CYP7A1 decreased in HFD‐fed mice exposed to antibiotics in early life (*p* = 0.022) (Figure 4G), which indicated the modulation of microbiome perturbations on inherent metabolic capacity which drives the phenotypic HFD resistance.^36^, ^37^, ^38^

### 2.4 Multiomic analysis revealed the disturbance of glycerophospholipid metabolism and the lipotoxic effects of hepatic lysophosphatidyl choline (LPC) in triggering ER stress and apoptosis

**FIGURE 5:**
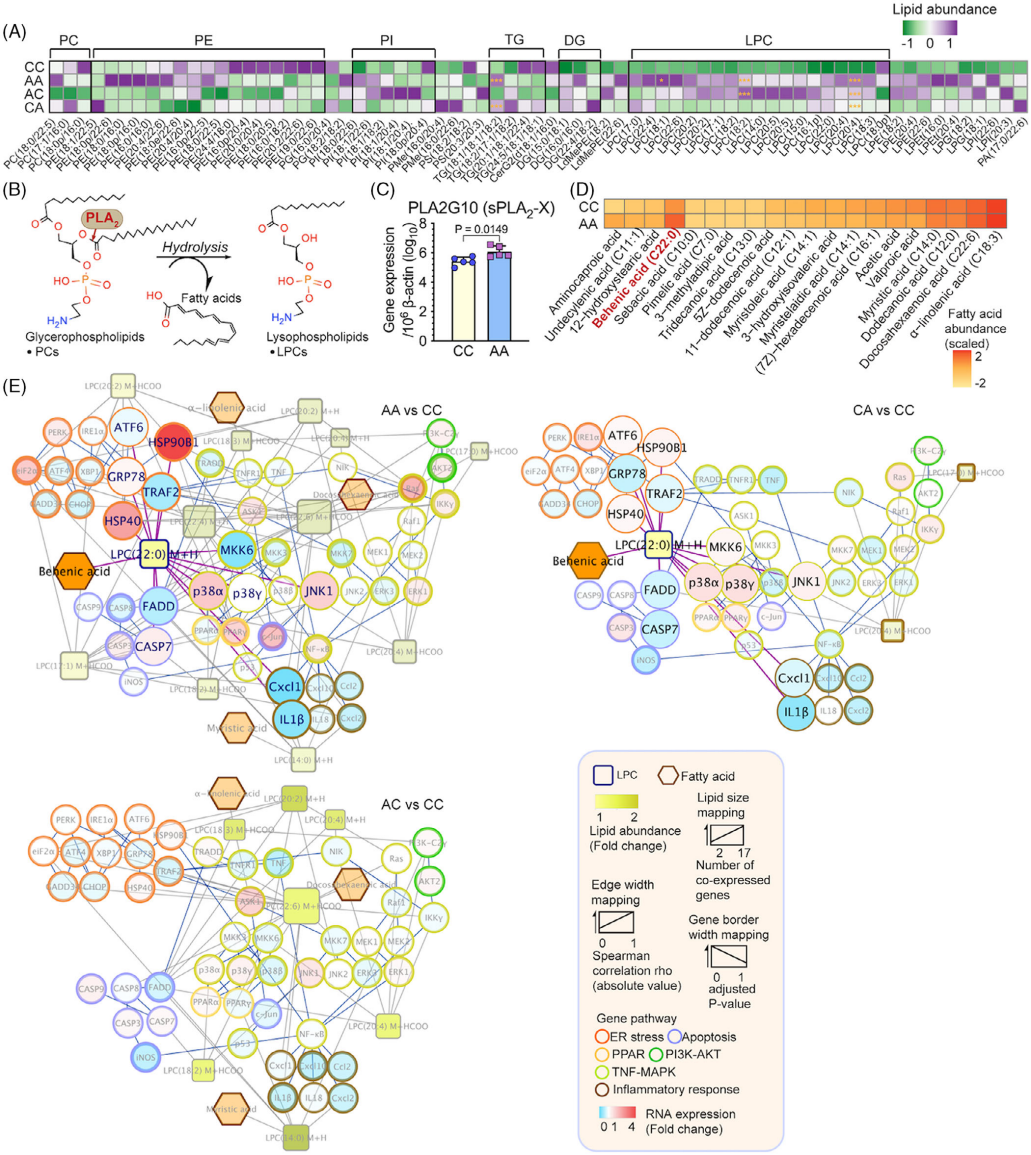
The multiomic interactive networks revealed *Parabacteroides*‐modulated glycerophospholipid metabolism and the cytotoxic effect of lysophosphatidyl choline (LPC). (A) Differentially expressed lipids [abundance ratio < 0.66 or > 1.2, and variable importance in projection (VIP) > 1]. Statistical significance was determined with two‐way ANOVA, *n* = 5, *p‐value  <  0.05, **p‐value  <  0.01. (B) A simplified presentation of the action of PLA_2_ cascade. PLA_2_ enzymes catalyze the hydrolysis of the sn‐2 ester bond in glycerophospholipids to produce free fatty acids and lysophospholipids. (C) Colonic expression of PLA2G10, a gene encoding group X secretory PLA_2_ (sPLA_2_‐X), in mice after HFD treatment (23 weeks old). (D) Heatmap showing the relative abundance of all the fatty acids identified through serum metabolomics. The graph was made based on the mean value (*n* = 3) of the measured abundance for each metabolite. (E) Networks profiling of the *Parabacteroides*‐associated lipid‐gene regulation. Differentially expressed (DE) LPCs (squares) that were also closely correlated with *Parabacteroides* were mapped to genes (circles) to draw an individual functional network showing the perturbations caused by different antibiotic exposures. All the interactions with LPC (22:0) were highlighted by an enlarged nodes and thick purple edges, while those interactions failing to show simultaneous co‐expression patterns with the respective fatty acids were hidden. The sizes of all the nodes are proportional to the number of interactions within each individual network. The color of the circle's outline was mapped to the biological pathways of the genes according to KEGG pathway. Gray edges indicate the associations between LPCs and genes. The edge thickness was drawn based on the value of the correlation coefficient. Blue edges indicated the regulatory paths between genes, which were derived from the literature. All the lipid, metabolite and gene abundance were obtained from pup mice after HFD treatment (23 weeks old).

To better understand the way in which the microbiome contributed to hepatic pathogenesis, we integrated serum metabolomics, hepatic lipidomics and transcriptomics to identify microbial markers associated with the dysregulated glycerophospholipid metabolism and the consequent lipotoxic effects and hepatocellular damage. A total of 71 differentially expressed lipids were identified among the different mice groups, with the majority of the lipids being glycerophospholipids (Figure 5A). Certain lipotoxic lipid groups (ER stress and apoptosis inducers), including diglycerides (DG) and LPCs,^25^, ^26^, ^39^ were significantly elevated in mice with early‐life antibiotic exposure (Figure S3A). Lysophospholipids, including LPCs, are known to be produced along with fatty acids by the Phospholipase A_2_ (PLA_2_) through hydrolyzing glycerophospholipids (Figure 5B). Interestingly, we observed a significant increase of gastric PLA2G10—a gene encoding group X secretory PLA_2_ (sPLA_2_‐X) (Figure 5C), which might have contributed to the dysregulated glycerophospholipid metabolism and LPC production.^40^ As previous studies suggested that LPS induces gastric permeability in parallel with the luminal secretion of PLA_2_ and LPCs,^40^ we examined the correlations between LPS‐expressing gut microbial taxa and the elevated lipid species (Figure S3B). *Parabacteroides*, an LPS‐producing Gram‐negative bacterium consistently enriched in both breastfed and HFD‐fed mice with postnatal antibiotic exposure, was positively correlated with LPC and DG levels (Figure S3B).

LPCs that were positively correlated with *Parabacteroides* were further investigated by integrating them into a lipid‐gene network (Table S2). Consistent with our hypothesis, the majority of the genes related to ER stress, apoptosis, and the phosphatidylinositol‐3‐kinase (PI3K)‐AKT pathway were positively correlated with the selected LPCs (Figure S3C). Consistent with prior studies,^41^, ^42^ the dominant function of these LPCs tended to be lipoapoptotic rather than pro‐inflammatory, as evidenced by their activation of executor caspases and transcriptional suppression of proinflammatory markers [e.g., C–C motif ligand 2 (CCL2), C‐X‐C motif chemokine ligand 1, 2, and 10 (CXCL1, CXCL2, and CXCL10), interleukin (IL)‐1β and IL‐18] (Figure 5E). Moreover, compared to antibiotic‐naive mice (CC), the serum level of the pro‐inflammatory cytokine tumor necrosis factor (TNF)‐α was significantly lower in HFD‐fed mice with early‐life antibiotic exposure (*p* < 0.05) (Figure S4). Associated with the presence of *Parabacteroides*, hepatocellular responses in HFD‐fed mice with both prenatal and postnatal antibiotic exposure (AA) were more largely perturbed (AA: 12 LPCs, 4 fatty acids and 153 correlations; CA: 3 LPCs, 1 fatty acid and 85 correlations; AC: 7 LPCs, 3 fatty acids and 109 LPC‐gene correlations) (Figure 5E).

As LPCs with different structures could function diversely in cholesterol biosynthesis and determining MASLD phenotype,^43^ we matched the LPCs with their cognate fatty acids (Figure 5D) to further pinpoint those originated from the dysregulated glycerophospholipid metabolism mediated by sPLA_2_‐X. Among all the matched pairs of fatty acids and LPCs, only behenic acid (22:0) and LPC (22:0) were simultaneously elevated in mice with antibiotic exposure and potentially generated through the action of sPLA_2_‐X (Figure 5E). The interactive networks included the activation of ER chaperons [heat shock protein (HSP) 40, HSP90B1], and apoptotic markers (p38, caspases).

### 2.5 *Parabacteroides* augmented LPS release and the pathogenesis of MASLD through metabolic perturbations and lipotoxicity effect in in vitro models

**FIGURE 6:**
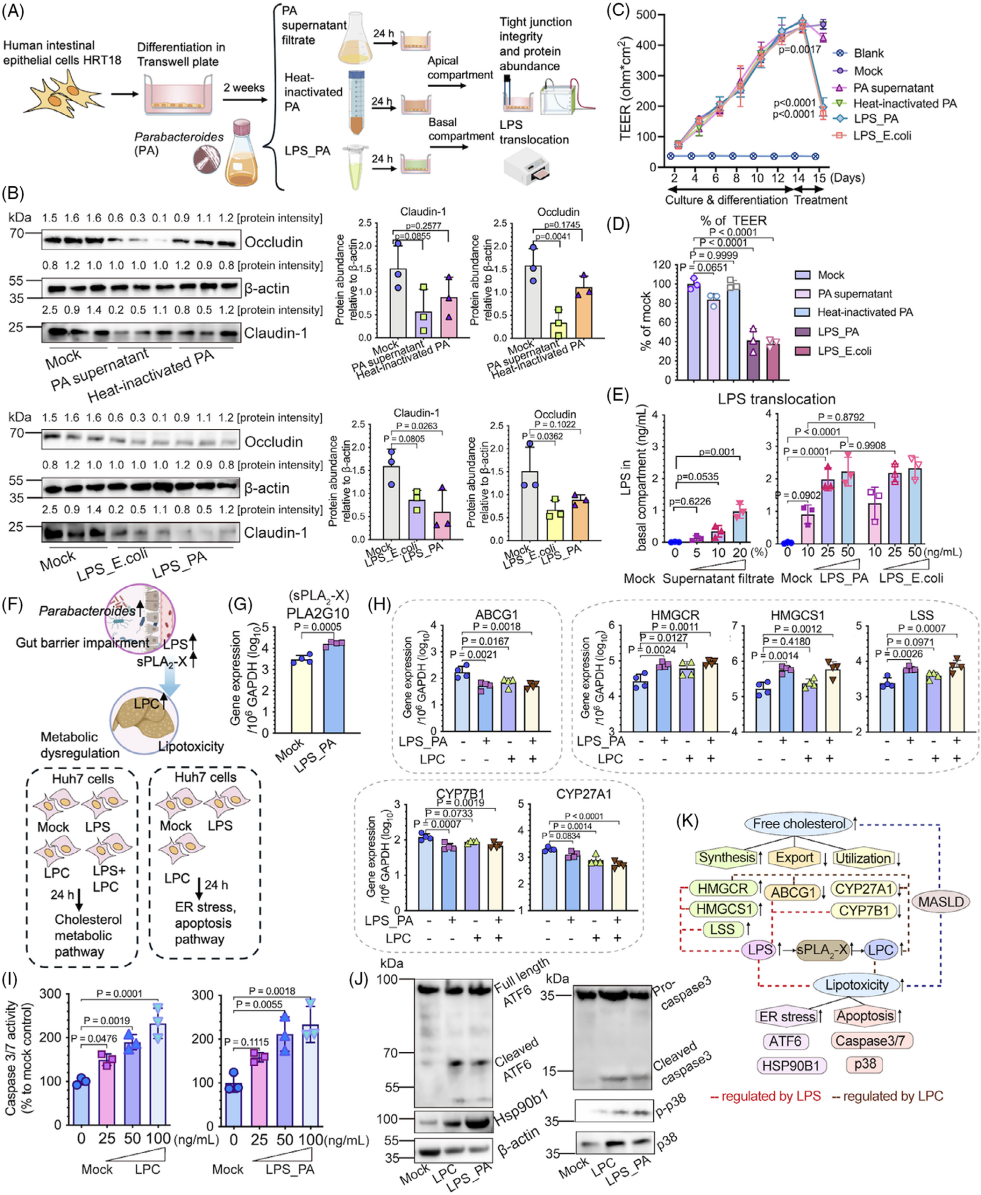
*Parabacteroides*‐induced gut barrier damage and LPS release led to hepatocellular apoptosis and the perturbation of cholesterol metabolic pathways in vitro. (A) Schematic overview of the experiments examining the functional components of *Parabacteroides* that affect gut barrier function and LPS release. (B) Western blot images and protein density quantification of tight junction proteins of HRT18 cells exposed to conditioned medium (Mock), PA supernatant filtrate, heat‐inactivated PA (upper panels), and LPS extracted from *E. coli* and PA (lower panels). The protein intensity was quantified by ImageJ. The normalized ratio was labeled above the respective band and was used for the quantification of occludin and claudin‐1 relative to β‐actin. Data represented as mean ± SEM, *n* = 3, one‐way ANOVA. (C, D) PA components‐induced integrity change measured by TEER. The final resistance values are expressed as: TEER = (*R* – *R_b_*) × *A*, where *R* is the measured resistance of the cells, *R_b_* is the resistance of the media blank control (no cells), and *A* is the area of the filter (1.12 cm^2^). (E) LPS translocation measured by the amount of LPS in the basal compartment. Cells treated with 20% of conditioned medium or DMSO served as mock controls. (F) Graphical presentation of the metabolic dysfunctions and hepatocellular lipotoxicity modulated by *Parabacteroides* through the gut‐liver axis. Huh7 cells were treated with DMSO, LPS_PA (50 ng/mL), LPC (22:0) (50 ng/mL), or a combination of LPS and LPC (50 ng/mL vs. 50 ng/mL) for 24 h and harvested to measure the changes of gene markers of cholesterol metabolic pathways and cell death pathways. (G) The expression of PLA2G10, a gene encoding sPLA_2_‐X in HRT18 cells with or without LPS_PA treatment. Data represented as mean ± SEM, *n* = 4, Student's *t*‐test. (H) Expression of gene markers in cholesterol synthesis (HMGCR, HMGCS1, LSS), export (ABCG1) and utilization (CYP7B1, CYP27A1) pathways in Huh7 cells treated with DMSO, LPS_PA (50 ng/mL), LPC (22:0) (50 ng/mL), or a combination of LPS and LPC (50 ng/mL vs. 50 ng/mL). Data represented as mean ± SEM, *n* = 4, one‐way ANOVA. (I) Caspase cleavage induced by LPS_PA and LPC (22:0). Data represented as mean ± SEM, *n* = 4, one‐way ANOVA. (J) Western blot images showing the activation of ER stress (ATF6 cleavage and Hsp90b1 upregulation), and apoptosis (phosphorylation of p38 and caspase 3 cleavage) in Huh7 cells treated with DMSO, LPS_PA (50 ng/mL) or LPC (22:0) (50 ng/mL). (K) Schematic diagram showing the two pathogenic processes of MASLD. Endotoxemia and the accumulation of cytotoxic lipids including LPC dysregulate the synthesis, export and utilization of cholesterol, and trigger ER stress and apoptosis following the primary lipid accumulation in liver. PA, *Parabacteroides distasonis*; TEER, transepithelial electrical resistance. Icons from (A) and (F) were made using BioIcons.

To investigate how overblooming of *Parabacteroides* following early‐life antibiotic exposure exacerbated the pathogenesis of MASLD, we first tested the functional components of *Parabacteroides* that affect gut barrier function and LPS release. To that end, filtered *Parabacteroides distasonis* (PA) supernatant, heat‐inactivated PA, and LPS extract of PA (LPS_PA) were added into the differentiated human intestinal epithelial cells HRT18 in Transwells to compare their effect on abundance and integrity of tight junction proteins (Figure 6A). Compared to heat‐inactivated PA, PA supernatant more dramatically decreased the expression of Claudin‐1 (not significantly) and Occludin (*p* = 0.0041) (Figure 6B, upper panels). LPS_PA significantly decreased the expression of Claudin‐1 (*p* = 0.0263) (Figure 6B, lower panels). Of note, LPS_PA induced the largest increase of monolayer permeability (over 58.5%, *p* < 0.0001) amongst all PA components as measured by transepithelial electric resistance (TEER) (Figure 6C and D), and allowed more efficient LPS translocation thereafter (Figure 6E). These results strongly hinted that LPS of PA and those released form dampened gut barrier and further facilitated the LPS translocation. Intriguingly, these effects exerted by LPS extract of PA seemed to be comparable with those extracted from *E. coli* (Figure 6B‐E).

The consequential effects of LPS_PA on glycerophospholipid metabolism and LPC production were examined (Figure 6F). Conform to our hypothesis, LPS_PA treatment increased the transcriptomic expression of sPLA_2_‐X (PLA2G10) in HRT18 cells (Figure 6G), suggesting an enhanced glycerophospholipid hydrolysis and LPC production.^40^ Therefore, we proceeded to investigate the subsequent metabolic perturbations and hepatocelluar damages caused by the overproduction of LPS and LPCs. Our results showed that individually and combinatorially administered LPS_PA and LPC (22:0) overall promoted cholesterol accumulation in Huh7 cells, while there exists a discrepancy of their target genes (Figure 6H). Individually and combinatorially administered LPS_PA and LPC (22:0) both upregulated HMGCR and downregulated ABCG1 to augment cholesterol synthesis and limit cholesterol export (Figure 6H). Interestingly, HMGCS1, LSS and CYP7B1 were only regulated by LPS_PA, while CYP27A1 only responded to LPC (22:0) treatment (Figure 6H). While LPS extracted from *E. coli* could also perturb cholesterol metabolism, it might function through PLA2G10‐inpdependent pathways (Figure S6).

The “double‐hit” hypothesis proposed massive hepatocellular damage as a “second hit” of MASLD pathogenesis, following the primary lipid accumulation.^44^ Our results showed that LPS_PA and LPC (22:0) treatment induced apoptosis in Huh7 cells at similar levels (Figure 6I and J), specifically by triggering ATF6 cleavage and elevating Hsp90b1, followed by the phosphorylation of p38 and the cleavage of the executor caspase (Figure 6J). Collectively, we demonstrated that overblooming of *Parabacteroides* could potentially dampen gut barrier functions and induce endotoxemia in an in vitro model. The excessive lipid metabolite LPC and circulating LPS coordinately disrupted cholesterol metabolism and induced ER stress and apoptosis in hepatocytes (Figure 6K).

## 3 DISCUSSION

To our knowledge, this is the first study using a life‐course animal model to show that early‐life antibiotic exposure can perturb the gut microbiota and subsequently promote metabolic dysfunction and hepatic steatosis, leading to the development of MASLD. Postnatal antibiotic exposure was a critical time period during which long duration of exposures resulted in more prominent disruptions in intergenerational gut microbiota transfer and development of hepatic steatosis. The observed endotoxemia and metabolic dysfunctions following early‐life antibiotic exposure can be mechanistically explained by the overblooming of gut *Parabacteroides* and the subsequent accumulation of LPCs with associated pathological pathways such as ER stress and apoptosis in hepatocytes. Moreover, our study showed that early‐life antibiotic exposure could overcome the inherent metabolic capacity of BALB/c mice and aggravate metabolic dysfunction and hepatic steatosis.

Previous studies have shown that microbiota dysbiosis induced by low‐dose penicillin in early life, rather than the antibiotic itself, can cause long‐lasting metabolic consequences and excessive adiposity.^28^, ^45^ Indeed, we found that the perturbations in the gut microbiota of offspring caused by maternal antibiotic administration could be long‐lasting. In dams, antibiotic administration only transiently decreased the α‐diversity of the gut microbiota, which recovered upon antibiotic withdrawal. By contrast, in pups, the decrease in gut microbiota α‐diversity from early‐life antibiotic exposure lasted longer. In humans, the resilience of the gut microbiome against antibiotic perturbation is influenced by the state of the microbiome, diet, exposure duration, route of administration, and spectrum of antibiotic activities.^46^ While the gut microbiota of healthy adults tends to be resilient to short‐term broad‐spectrum antibiotic interventions, those of infants respond more dramatically to antibiotic perturbations.^46^, ^47^, ^48^ Subject to factors such as antibiotic dose and breastfeeding mode, the microbiome of infants can fail to completely recover for over two years after an antibiotic intervention in the first year of life.^32^, ^46^ Long‐term dysregulation of childhood microbiome predisposes to multiple diseases including asthma and overweight/obesity, alongside persistent metabolic consequences.^49^, ^50^ Although the effect of penicillin on the gut microbiota has been arbitrarily considered weaker than macrolides,^49^, ^51^ our results highlight that it can still have long‐term impacts on offspring following maternal administration during early life. The preexisting metabolic disorders after antibiotic exposure may potentiate into a long‐term advanced disease status with synergistic impacts of adverse environmental factors.

Compared to prenatal antibiotic exposure, postnatal antibiotic exposure disrupted intergenerational gut microbiota transfer fidelity and altered early‐life microbiota onset to a greater degree (Figures 2 and 3). In pups exposed to antibiotics postnatally, certain beneficial microbiota such as *Lactobacillus* ^52^ and *Rikenellaceae* ^28^ were not transferred from dams to pups and they were continuously deficient until adulthood despite several diet shifts (Figures 2A and 3B). The theory of “disappearing microbiota” has proposed an association between the gut microbes lost during vertical transmission with the epidemics of chronic diseases such as obesity.^53^ The substantial impact of indirect antibiotic exposure on the offspring's gut microbiome composition could be attributable to the postnatal period being a critical period for gut microbiota development: the microbiota is seeded from mothers at birth. In human studies, intrapartum postnatal administration of antibiotics tended to more profoundly impact on infant gut colonization, leading to a decreased diversity, a decreased proportion of the phyla *Actinobacteria* and *Bacteriodetes* and increased *Proteobacteria*. By contrast, these taxonomic compositional changes and the species richness were compromised or not significant in infants born by mothers with prenatal antibiotics exposure, although potential biases on feeding mode, route of delivery, gestational age and the type and duration of antibiotic use could not be minimized in these studies.^9^ Early‐life antibiotic exposure could affect breast milk microbiome composition and, by extension, the offspring's gut microbiota.^54^ Breastfeeding helps develop a healthy gut microbiome and reduces the risk of childhood overweight.^55^ There should therefore be greater consideration into the prescription of penicillin for lactating women, as this may have eliminated the benefits of maternal breast milk and predisposed infants to metabolic dysfunction in our mice model.

We profiled the etiology of gut microbiota‐associated metabolic disorders and mechanistically linked the enrichment of *Parabacteroides* in pups with postnatal antibiotic exposure in MASLD pathogenesis (Figures 3, 4, 5, 6). Studies on *Parabacteroides* highlighted its dichotomous role in the development of metabolic disorders, depending on the administration context and strain‐to‐strain differences.^56^, ^57^, ^58^ In our mice model, the bloom of opportunistic bacteria such as *Enterorhabdus* and *Parabacteroides* following prolonged usage of antibiotics in early life, possibly out‐competed the developmentally‐beneficial microbial taxa, causing gut dysbiosis and systemic endotoxemia (Figures 3D and 4A). It has been shown that low dose LPS (as little as 1 ng/10 g of body weight) could significantly increase the lipid generation and secretion as well as the serum triglyceride in mouse models.^59^ In experimental and clinical studies, the increase of *E. coli* derived LPS has been linked to in MASLD and liver damage by inducing macrophage and platelet activation through the TLR4 pathway.^60^, ^61^ This LPS‐associated metabolic dysfunctions has also been observed in our study: LPS derived from the bloom of *Parabacteroides*, directly modulated cholesterol metabolism and hepatic apoptosis pathways, meanwhile elevates sPLA_2_‐X and caused the accumulation of cytotoxic LPCs (Figure 6). Specifically, our multiomic analysis pinpointed LPC (22:0) as a death effector and key driver of MASLD development (Figures 5 and 6). Given the complexity of the onset of metabolic illness following early‐life antibiotic exposure, the microbial drivers including *Parabacteroides* should be comparatively studied. A detailed regulation of microbial taxa and systemic LPS pool could be profiled based on bacteria genomic and transcriptomic analysis of genes controlling the biosynthesis of LPS, to map the microbiome perturbations and LPS‐associated metabolic dysfunctions.

Our study also revealed the complexity and crosstalk of metabolic dysregulations and hepatocyte damage in MASLD pathogenesis. Excessive lipids generate lipotoxicity effect and a global disturbance of hepatocellular homeostasis. Subsequently aberrant unfolded proteins accumulate within the ER membranes, causing the “ER stress.” Persistent ER stress leads to unfold protein responses (UPR) and could ultimately progress into apoptosis through three stress sensors, namely, inositol‐requiring enzyme 1 alpha (IRE1α), protein kinase RNA‐like ER kinase (PERK), and activating transcription factor 6 (ATF6).^62^ Hepatocyte apoptosis is the predominant cell death pathway in nonalcoholic steatohepatitis.^63^ As ER plays an important role in regulating triglyceride and cholesterol biosynthesis,^64^ UPR and apoptosis also implicate abnormal lipid synthesis.^65^ In our study, postnatal and prenatal antibiotic exposure differentially activated the ER stress and apoptosis pathways to various extents. This could be explained by the various LPCs generated and their individual functions.^66^ In consensus with previous reports,^41^, ^42^, ^67^, ^68^, ^69^, ^70^ we confirmed the role of LPS_PA and LPC (22:0) in triggering cell death, and augmenting MASLD pathogenesis^23^ via disturbing the production, utilization and transport of cholesterol (Figure 6H‐J). Direct injection of a L‐α‐LPC to ICR mice has been shown to induce lobular hepatitis and apoptosis at 3 days post treatment.^41^ In that study, the types of available free fatty acids and inflammatory cytokines were assumed to regulate the conversion of DG into either LPC or triglyceride.^41^ Here in our early‐life antibiotic exposure mice model, a predominant DG→PC→LPC pathway over DG→TG pathway, alongside the defects in systemic immune development, may have shifted hepatic cell fate from survival (inflammation) toward death. Further work will be required to address the differential functions of the LPCs and the cognate fatty acids following early‐life antibiotics exposure, especially in clinical settings.

Our antibiotic exposure model could be further improved in generalizability, by further investigating the timing, duration, and class of antibiotics that could differentially affect metabolic outcomes.^9^, ^71^ *Enterorhabdus* and *Parabacteroides* have been reported to coordinate with LPCs and damage the epithelial barrier in a colitis mice model.^72^, ^73^ Our study also showed similar co‐expression patterns between these two taxa with LPC molecules under a synergistic effect of antibiotics and HFD (Figure S3B). While we elucidated *Parabacteroides*‐mediated hepatocellular responses, further comparative and functional investigations on *Enterorhabdus* are needed. A total of 13 *Parabacteroides* species, among 20 culturable species, have been identified as inhabitants of the human intestine^74^ and different species are associated to different diseases.^75^, ^76^ Therefore, species‐ or strain‐level identification of *Parabacteroides* and their mechanistic contributions to the development of liver steatosis are warranted.

The mouse strain was used given their unique metabolic capacity driven by genetic factors, marked by the irresponsiveness of hepatic CYP7A1 to HFD treatment^36^, ^37^, ^38^; this allows us to evaluate whether early‐life gut dysbiosis could modulate the intrinsically silenced metabolic processes. Here, we observed that antibiotic‐induced gut dysbiosis in early life suppressed hepatic CYP7A1 in BALB/c mice, suggesting that microbiome can overcome the inherent metabolic capacity driven by the genetic determinants. However, further investigations using transgenic mice and other CYP7A1‐irresponsive strains (i.e., NZB and 129/J) are needed to confirm this generalizability.^36^, ^37^, ^38^ Moreover, although the functional role of the CYP7A1 gene in humans has been reported,^77^, ^78^ particularly its deficiency in hyperlipidemia,^78^ the generalizability of our findings to humans needs further investigation.

Our study has several strengths. While numerous observational studies have investigated the long‐term health outcomes of neonates following prenatal and postnatal antibiotic exposures, complex factors that interact with microbiome in early life continue to hinder our understanding of microbiome‐driven metabolic outcomes in humans.^6^, ^9^, ^71^ In addition, the heterogeneity among studies, different outcome measurements, and microbiota detection methods hamper the generalizability of observations.^9^ Our study is the first of its kind to trace gut microbiota composition throughout the entire infancy period using an animal model and showed that microbiota‐driven mechanisms aggravates metabolic dysfunction in early life, by eliminating the major confounding factors such as delivery mode and maternal diet. While effective stewardship for early‐life antibiotic treatments is still an obstacle, we anticipate that our study will help inform clinical practices and health policies regarding the collateral damage of maternal antibiotic administration in early life, especially on the risk of developing MASLD. Efforts should be made toward a better use of other detection tools for bacteremia in the clinical practice, in order to avoid any prolonged antibiotic administration. Since there are currently no effective treatments for MASLD, our findings highlight potential prognostic biomarkers and the importance of early interventions using gut microbiota‐targeted strategies. The integrated microbiome and multiomic framework will allow identifying more central hubs and checkpoints that could be used as preventive or therapeutic targets for MASLD.

## 4 METHODS

### 4.1 Mouse strain, husbandry, and treatment

Pairs of 8‐week‐old conventional BALB/c mice were obtained and maintained under specific pathogen‐free (SPF) facilities of The Centre for Comparative Medicine Research (CCMR) to breed the study group litters. Standardized housing conditions were maintained with a 12‐h light/dark cycle and ad libitum access to diet and water throughout the experiment. Dams were randomly assigned into four groups according to whether they were kept conventionally (C) or with antibiotic administrations (A): (1) with antibiotic only prenatally (AC), (2) with antibiotic only postnatally (CA), (3) with antibiotic both prenatally and postnatally (AA), and (4) conventionally from prenatal to postnatal periods (CC). Penicillin V was administered to the assigned dams via the drinking water at 0.25 mg/mL in their late pregnancy (the last week prior to laboring), late lactation period (the last week prior to weaning), or during both pregnancy and the breastfeeding period. The antibiotic supplementation was calculated by adjusting the therapeutic doses of penicillin based on comparable peak serum concentrations between a therapeutic human dose (250 mg) and an oral dose (40 mg/kg) used on animal models.^79^, ^80^, ^81^ Water was refreshed every 3 days for the duration of the experiment. Dams without penicillin V treatment served as a control group. Pups were weaned at Day 21 and separated by sex to retain only males. All retained pups were then assigned into the four dam groups per their antibiotic exposure (AA: pups born by dams with both prenatal and postnatal antibiotic exposure; AC: pups born by dams with only prenatal antibiotic exposure; CA: pups born by dams with only postnatal antibiotic exposure; CC: pups born by dams without maternal antibiotic exposure). All groups of pups underwent a 10%  kcal fat standard chow diet (CD) period for five weeks to obtain identical weights, before shifting to a 45%  kcal fat HFD until the end of the experiment. Their body weight was monitored throughout the experiment. Ethical approval for animal experiments were obtained from the Committee on the Use of Live Animals in Teaching and Research (CULATR 4852‐18).

### 4.2 Cell culture

The human intestinal epithelial cells HRT18 were maintained in RPMI‐1640 (Gibco) medium supplemented with 10% fetal bovine serum (FBS) and 100 U/mL penicillin/streptomycin. Human hepatoma cell line Huh7 was cultured in Dulbecco's modified Eagle's medium (DMEM, Gibco) containing 2 % of human serum (Merck) and 100 U/mL penicillin/streptomycin since human serum cultured Huh7 cells has been reported to demonstrate more physiological relevant features in terms of fatty acid metabolism.^82^

### 4.3 Fecal DNA extraction, 16S rRNA sequencing, and microbiome analysis

Fecal samples were collected from adult and infant mice, weighed, and stored with DNA/RNA Shield‐fecal preservation buffer (Zymo Research) at −80°C prior to DNA extraction. Genomic DNA was extracted from fecal samples using QIAamp PowerFecal DNA Kit (Qiagen) according to manufacturer's instructions. Resulting DNA templates were subjected to 300  bp paired‐end double‐index Illumina sequencing approaches to target V3‐V4 hypervariable regions of the 16S rRNA gene. Raw 16S rRNA sequences were analyzed using the QIIME2 pipeline.^83^ Within‐community diversity (α‐diversity) was calculated by Chao1 and Observe ASVs. Between‐sample differences of microbiota diversity (β‐diversity) were assessed by Bray–Curtis dissimilarity index, tested by Permutational Multivariate Analysis of Variance (PERMANOVA) and visualized by PCoA and CCA. The LEfSE was used to identify bacterial taxa altered by early‐life antibiotic exposures. We considered taxa with LDA score > 2 and *p* < 0.05 to be statistically significantly differential between groups. Associations between microbiome composition and lipids were tested using Spearman's correlation coefficient.

### 4.4 Serum lipopolysaccharides, lipid biochemistry, and cytokine quantification

Sera were obtained from whole blood samples to examine LPS (Mouse LPS ELISA Kit, Cusabio), total cholesterol triglycerides (Cholesterol LiquiColor® Test, Stanbio), HDL‐C (Direct HDL‐Cholesterol LiquiColor® Test, Stanbio), and LDL‐C following the manufacturers’ respective protocols. Mice serum cytokine levels, including TNF‐α, IL‐2, IL‐6, IL‐9, IL‐13, IL‐17A, IL‐17F, and IL‐22 levels, were measured using LEGENDplex™ Mouse Th Cytokine Panel (Biolegend) according to the manufacturer's instructions. Data was acquired on an Attune™ NxT Acoustic Focusing Cytometer and analyzed with the LEGENDplex™ Data Analysis software (Biolegend) using a five‐parameter logistic curve to derive the standard curve. Cytokine levels were calculated based on their respective standard curve and presented as values over the detection limit.

### 4.5 Histopathological examination

Mice liver was fixed in 10% neutral‐buffered formalin for hematoxylin and eosin (H&E) staining. Hepatic lipid content was determined using frozen sections stained with Oil Red O. Images were acquired on a Leica DM3000 microscope.

### 4.6 Western blot

Expressions of claudin‐1 and occludin were quantified by western blot analysis. Mouse anti‐claudin‐1 antibody (Santa Cruz Biotechnology, sc‐166338), rabbit anti‐occludin antibody (Abcam, ab167161), mouse anti‐ATF6 antibody (Abcam, ab122897), rabbit anti‐caspase‐3 antibody (Abcam, ab32351), rabbit anti‐Hsp90b1 (GRP94) (Abcam, ab238126), phospho‐p38 MAPK antibody (Thermo Scientific, 36–8500), p38 MAPK antibody (Thermo Scientific, p38‐3F11), and mouse anti‐β‐actin antibody (Abcam, ab8226) were used as primary antibodies for the detection of each respective target protein. Goat anti‐rabbit IgG (H + L) horseradish peroxidase (HRP) conjugated antibody (Thermo Scientific, 656120) and goat anti‐mouse IgG (H + L) HRP antibody (Thermo Scientific, 626520) were used as secondary antibodies in the western blot. For tissue sample processing, 2‐cm sections of mid‐jejunal intestine were harvested from each mouse and homogenized in 250 µL of ice‐cold RIPA‐buffer (Thermo Scientific, 89900) with protease inhibitor (Thermo Scientific, A32963) and ceramic beads (2.8 mm, Qiagen) by PowerLyzer 24 Homogenizer (Qiagen). This mixture was centrifuged at 12,000 × *g* for 10 min at 4 °C. The supernatant was collected for western blot analysis.

### 4.7 Transcriptomics, lipidomics, and metabolomics

For transcriptomics, mouse livers were homogenized in Trizol and total RNA was isolated for the construction of sequencing libraries. mRNA was enriched with Oligo (dT) magnetic beads and subsequently fragmented for cDNA synthesis. The stranded and polyA‐selected mRNA libraries were sequenced on the DNBseq™ system (Beijing Genomics Institute, China) using the single‐end 100 bp protocol. The clean data was mapped to the mouse reference genome (GRCm38) from the Ensembl database with STAR using default parameters.

For nontargeted lipidomics, 25 mg of mice liver was weighted, mixed with 800 µL of dichloromethane: methanol (3:1, v/v) and homogenized for lipid extraction. The homogenate was centrifuged, and the supernatant was taken for lyophilization and reconstitution. A LC‐MS system consisting of Waters 2D UPLC (waters) and Q Exactive high‐resolution mass spectrometer (Thermo Fisher Scientific). The raw files generated by liquid chromatography‐MS/MS detection were imported into LipidSearch v.4.1 (Thermo Fisher Scientific) for lipid identification and peak alignment.

Targeted quantitative metabolomics of serum samples was performed using the HM700 metabolome detection kit (Beijing Genomics Institute, China) according to previously recorded methods.^84^ Metabolites extracted from the mouse serum were analyzed using Waters UPLC I‐Class Plus equipped with QTRAP 6500 Plus (SCIEX) through the Multi Reaction Monitoring, multiple reaction detection scanning (MRM) mode. Metabolite concentration was calculated by the Skyline software (MacCoss Lab Software) using default parameters for each MRM Transition's automatic identification and integration, assisting with manual inspection. The content of each substance (µmol/g) = *C* × *A*/*m*; *C* = the concentration value obtained by bringing the integrated peak area of the target index in the sample into the standard curve (µmol/L); *A* = the dilution factor; *m* = the mass of the weighed solid sample (mg).

### 4.8 Integrated multiomics analysis

Differentially expressed (DE) transcripts between groups were analyzed using the EdgeR statistical package. Gene clustering was performed according to our in‐house pipeline to obtain the Gene Ontology (GO) terms and Kyoto Encyclopedia of Genes and Genomes (KEGG) pathways. Genes were screened by annotating their predominant functions to pathways that are closely related to MASLD pathogenesis, including the ER stress, apoptosis, inflammatory response, PI3K‐AKT, TNF‐MAPK, and peroxisome proliferator‐activated receptor (PPAR) signaling pathways. Statistical analysis for lipidomics was performed on metaX.^85^ To screen for DE lipid molecules, we evaluated the variable importance in projection (VIP) values of the first two principal components of the PLS‐DA^86^ and fold changes of lipid abundance. Differentially expressed lipids were defined as those with an abundance ratio of < 0.66 or > 1.2 and VIP > 1. Spearman's correlation analysis was conducted to identify the co‐expression patterns between dysregulated bacterial taxa, DE lipids, and genes that had been functionally screened.

To investigate microbiota‐mediated responses to HFD, fold changes of the genes and LPCs were calculated by comparing their expressions in groups exposed to antibiotics with those in the control group under HFD. Strong positive correlations (Spearman's correlation coefficient > 0.3) between microbiota and lipidomic markers and strong correlations (Spearman's correlation coefficient > 0.4 or <‐ 0.4) between lipidomic and transcriptomic markers were selected and merged to construct an integrated network. Gene‐gene interactions were identified in the literature, mapped with the fold changes, and integrated to show microbiota‐mediated signaling events. Based on the principle of the hydrolysis process of glycerophospholipid mediated by PLA_2_, LPCs were matched to their cognate fatty acids to further pinpoint those simultaneously elevated in mice with antibiotic exposure.

### 4.9 Bacterial culture and LPS extraction

BLAST search of 31 ASV sequences of *Parabacteroides* (lengths ranging from 463 to 469 nucleotides) yielded highest identity (99.14%) to *Parabacteroides distasonis*. To prepare for representative functional components of *Parabacteroides*, in‐house isolated *Parabacteroides distasonis* (PA) were anaerobically cultured in Cooked Meat Medium at 37°C overnight, to obtain cell‐free supernatant filtrate, heat‐inactivated bacteria, and LPS extract. The overnight culture (2 × 10^8^ CFU/mL, 10 mL) was centrifuged at 6000 rpm for 15 min, to collect either filter‐sterilized culture supernatant filtrate or sedimented bacteria. Bacterial supernatant filtrate (of around 5 ng/µL of LPS) was neutralized to pH 7.0 before use. To prepare for the heat‐inactivated bacteria, the bacterial pellet was washed with PBS, sonicated for 90 s, and boiled for 10 min. The total volume was brought to 10 mL with PBS. The LPS was isolated from the bacteria pellet using LPS Extraction Kit (Boca Scientific) according to the manufacturer's instructions. The concentration of LPS was determined using LAL Chromogenic Endotoxin Quantitation Kit (Thermo Fisher). The LPS extract of *E. coli* was prepared in the similar way.

### 4.10 Transepithelial electrical resistance (TEER) and LPS translocation assay

HRT18 cells were seeded onto Transwell inserts (0.4 µm pore size, 1.12 cm^2^, Costar) at a density of approximately 2 × 10^5^ cells per cm^2^ and cultured for 14 days to form the tight junctions. The growth medium was supplied to both the apical and basal compartments and was replenished every other day. A Millicell ERS‐2 Volt‐Ohm Meter (EMD Millipore) was used to measure the TEER every other day to monitor the formation of the tight junctions. On the day of treatment, a final concentration of 20 % bacterial supernatant filtrate (of around 5 ng/µL of LPS), 20 % heat‐inactivated bacterial homogenate and 50 ng/mL of LPS extract was added into the apical compartment respectively. Mock treatment group was set by supplying with an equal volume of bacteria culture medium. TEER was measured at 24 h post treatment to compare the impact of each bacterial product on the epithelial barrier integrity.

To assess the amount of LPS translocated into the basal compartment of the Transwell system, serially diluted bacterial supernatant filtrate and extracted LPS was added into the apical compartment. Cells treated with 20% of conditioned medium or DMSO served as mock controls. At 24 h post treatment, medium in the basal compartment was harvested for LPS quantification.

### 4.11 Quantitative reverse transcription polymerase chain reaction (RT‐qPCR)

Cells were lysed in RL buffer and extracted with the MiniBEST Universal RNA Extraction Kit (TaKaRa). RT and qPCR were performed with Transcriptor FirstStrand cDNA Synthesis Kit and LightCycler 480 master mix (Roche). Primers used in RT‐qPCR reactions were listed in Table S3. Human gene expression was normalized to human glyceraldehyde‐3‐phosphate dehydrogenase (GAPDH) mRNA, whereas the mouse gene expression was normalized to mouse β‐actin mRNA. Relative gene expression was quantified using the CT (2^− ΔΔCT^) method.

### 4.12 Caspase assay

Huh7 cells were seeded in a Corning® 96 well plate (Cat. CLS9102) and treated with LPC (22:0) (Avanti Polar Lipids) or LPS_PA under serial dilutions for 24 h. The Caspase‐Glo‐3/7 assay (Promega) was used to quantify the activation of executor caspases including caspase‐3 and caspase‐7. Cells were lysed with the Caspase‐Glo‐3/7 reagent at the designated time points, followed by measuring the luminescence signal with the Vector X3 multilabel plate reader (PerkinElmer).

### 4.13 Statistical analyses

Statistical comparisons between different groups were made by one‐way ANOVA, two‐way ANOVA, or Student's *t*‐test using GraphPad Prism (GraphPad Software). A *p* value < 0.05 was considered significant.

## AUTHOR CONTRIBUTIONS

H.M.T., X.Z., and D.C.L.C. conceptualized the study and designed the experiments, with the supervision of A.K., F.K.L.C., and S.C.N. X.Z. and D.C.L.C. performed the experiments with the crucial help of D.Z.Y.S., Y.T., and W.Z. X.Z., D.C.L.C., and J.Z. analyzed the data with the assistance of H.M.T. and Y.P. X.Z. and H.M.T. wrote the manuscript. H.M.T., M.K.L.W., A.M.H., J.Y. T., A.K., F.K.L.C., and S.C.N. critically reviewed the manuscript and provided intellectual input. All authors participated in discussing and revising the manuscript. All authors have read and approved the final manuscript.

## CONFLICT OF INTEREST STATEMENT

F.K.L.C. is Board Member of CUHK Medical Centre. He is a cofounder, nonexecutive Board Chairman and shareholder of GenieBiome Ltd. He receives patent royalties through his affiliated institutions. He has received fees as an advisor and honoraria as a speaker for Eisai Co. Ltd., AstraZeneca, Pfizer Inc., Takeda Pharmaceutical Co., and Takeda (China) Holdings Co. Ltd. S.C.N. has served as an advisory board member for Pfizer, Ferring, Janssen, and Abbvie and received honoraria as a speaker for Ferring, Tillotts, Menarini, Janssen, Abbvie, and Takeda. S.C.N. has received research grants through her affiliated institutions from Olympus, Ferring, and Abbvie. S.C.N. is a scientific co‐founder and shareholder of GenieBiome Ltd. S.C.N. receives patent royalties through her affiliated institutions. F.K.L.C., S.C.N., H.M.T. are named inventors of patent applications held by The CUHK and MagIC that cover the therapeutic and diagnostic use of microbiome but have no potential relevant financial or nonfinancial interests to disclose. The other authors have no conflicts of interest to declare.

## ETHICS STATEMENT

This study involving mice was approved by the Committee on the Use of Live Animals in Teaching and Research (CULATR 4852‐18).

## Supporting information



Supporting Information

## Data Availability

Quality‐controlled 16S rRNA sequencing data and RNA sequencing data have been deposited into the European Nucleotide Archive under BioProjects PRJEB62481 and PRJEB63629. Lipidomics data is available at https://doi.org/10.6084/m9.figshare.23309462.v1. Any additional information required to reanalyze the data reported in this paper is available upon request.
