# Supplementary material for: Early‐life antibiotic exposure aggravates hepatic steatosis through enhanced endotoxemia and lipotoxic effects driven by gut Parabacteroides
Source: MedComm (2020). 2025 Feb 17;6(3):e70104. doi: 10.1002/mco2.70104 (PMC11832435; doi:10.1002/mco2.70104)
Supplement: Supplementary file 1 — Supporting Information [file MCO2-6-e70104-s001.pdf]

Early-life antibiotic exposure aggravates hepatic steatosis through enhanced endotoxemia and lipotoxic effects driven by gut *Parabacteroides*

Table of contents

Fig. S1……………………………………………………………………………………...1

Fig. S2……………………………………………………………………………………...2

Fig. S3…………………………………………………………………………….......3

Fig. S4…………………………………………………………………………….......4

Fig. S5…………………………………………………………………………….......5

Fig. S6…………………………………………………………………………….......8

Table S1……………………………………………………………….………..…....9

Table S2……………………………………………………………….……..………10

Table S3……………………………………………………………….……..………15

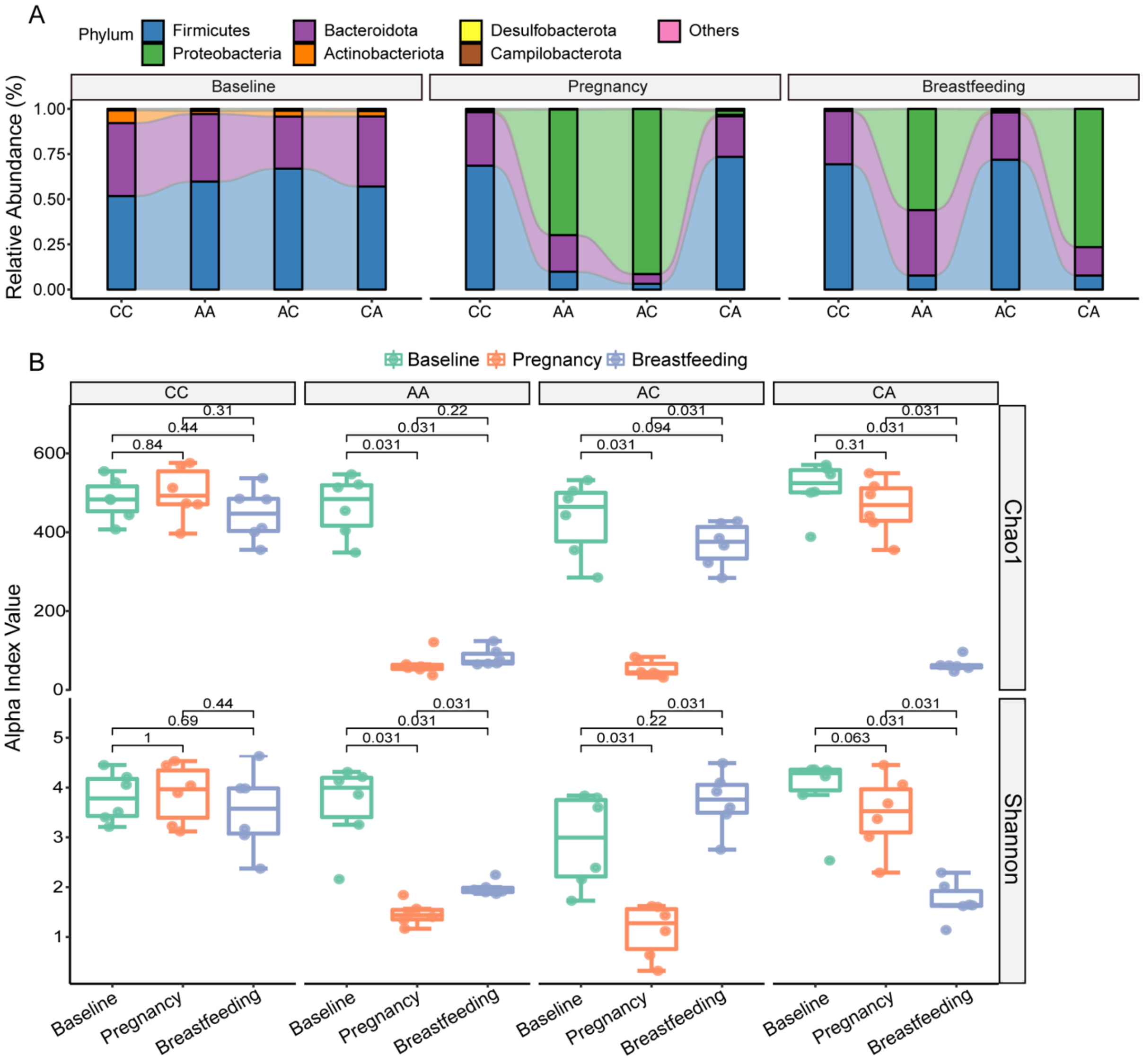

Fig. S1. (A) Compositional changes of the gut flora of dams. (B) α-diversity of the gut flora of dams calculated by Chao1 richness and Shannon index. Fecal samples collected from baseline, pregnacy and breastfeeding periods were on Day 12-13 of pregnancy, day 19-20 of pregnancy, and day 21 of breastfeeding, respectively.

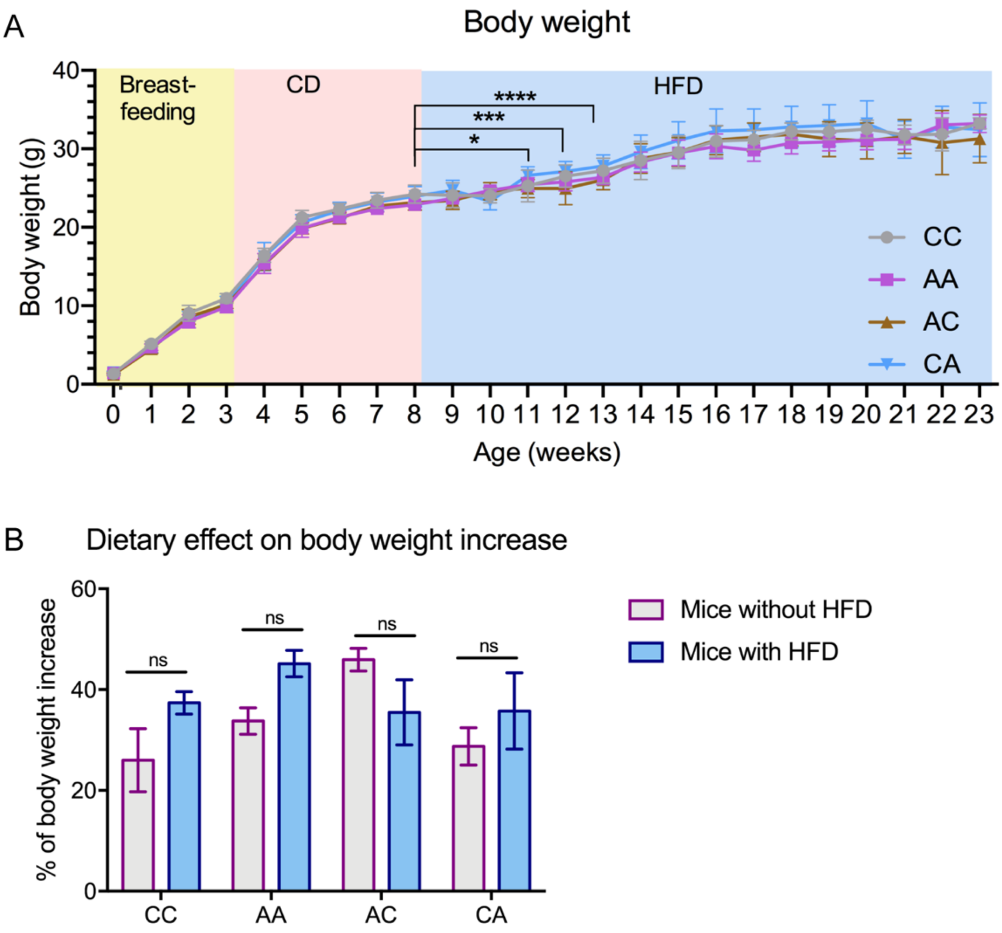

Fig. S2. (A) Body weight changes of pups (n=5 per group) from breastfeeding until the endpoint. (B) Dietary effect on the percentage of body weight increase. Two additional mice in CC, AA, AC, CA were continously seperated as control groups and supplemented with normal chow diet from birth until the end of the experiment. The body weight increase was calculated from the start of the HFD treatment (8-weeks old) to the end of the experiment (23-weeks old). All data represented as mean ± standard error of the mean. Statistical significance analyses were determined with two-way ANOVA. *P-value < 0.05, ***P-value < 0.001, ****P-value < 0.0001, ns: not significant. CD: chow diet period; HFD: high-fat diet period.

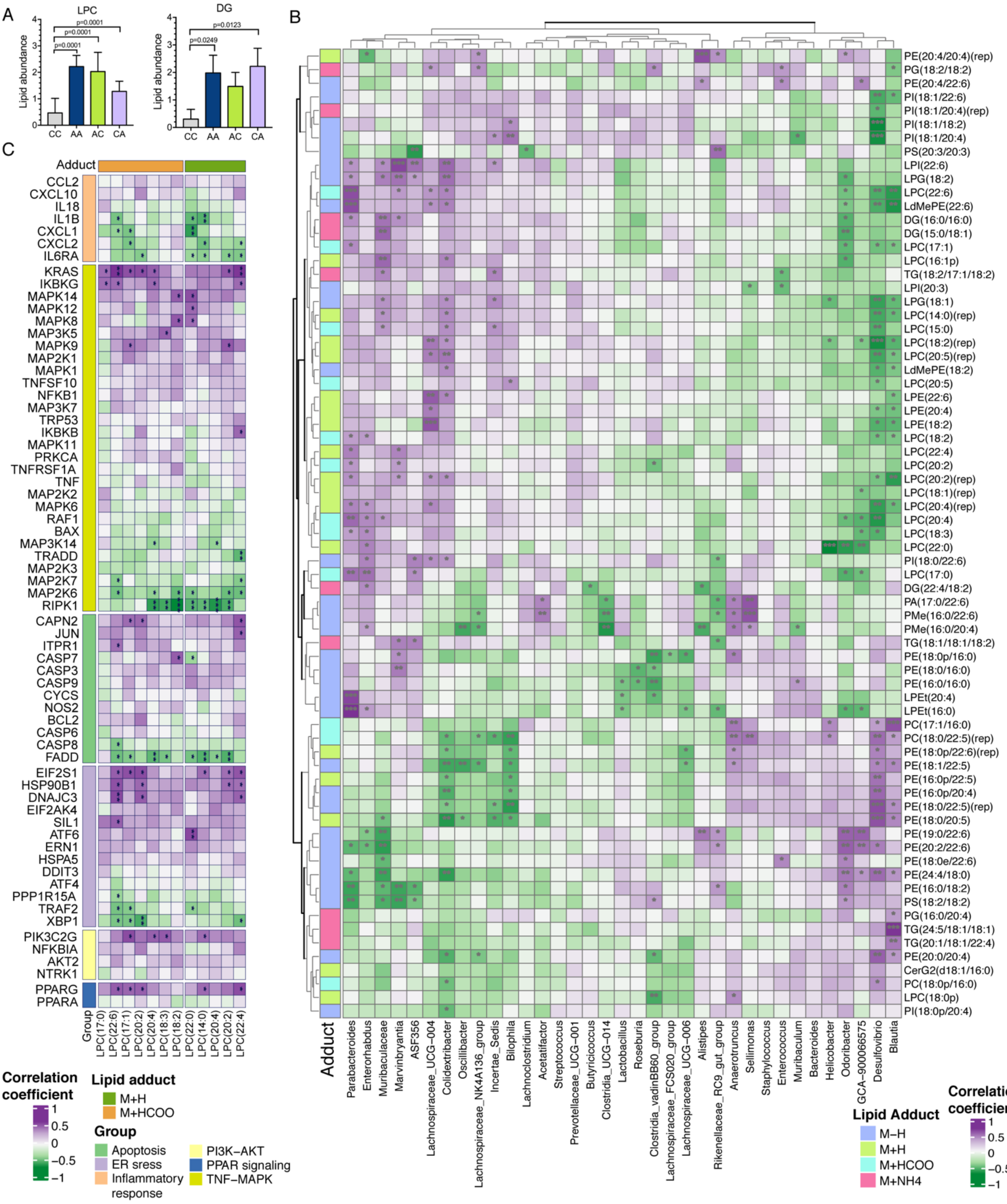

Fig. S3. (A) Relative abundance of hepatic lysophosphatidyl choline (LPC) and diglycerides (DG) identified by nontargeted lipidomics. Data represented as mean ± standard deviation, n = 5, one-way ANOVA. (B) Spearman's correlation coefficients between the antibiotics-altered bacteria taxa and the differentially expressed lipids. (C) Spearman's correlation coefficients between the differentially expressed LPCs and genes with annotated functions in the endoplasmic reticulum (ER) stress, apoptosis, inflammatory response, phosphatidylinositol-3-kinase (PI3K)-AKT, tumor necrosis factor (TNF)-mitogen-activated protein kinases (MAPK), and peroxisome proliferator-activated receptor (PPAR) signaling pathways. P value was calculated by wilcox text. *P-value < 0.05, **P-value < 0.01, ***P-value < 0.001.

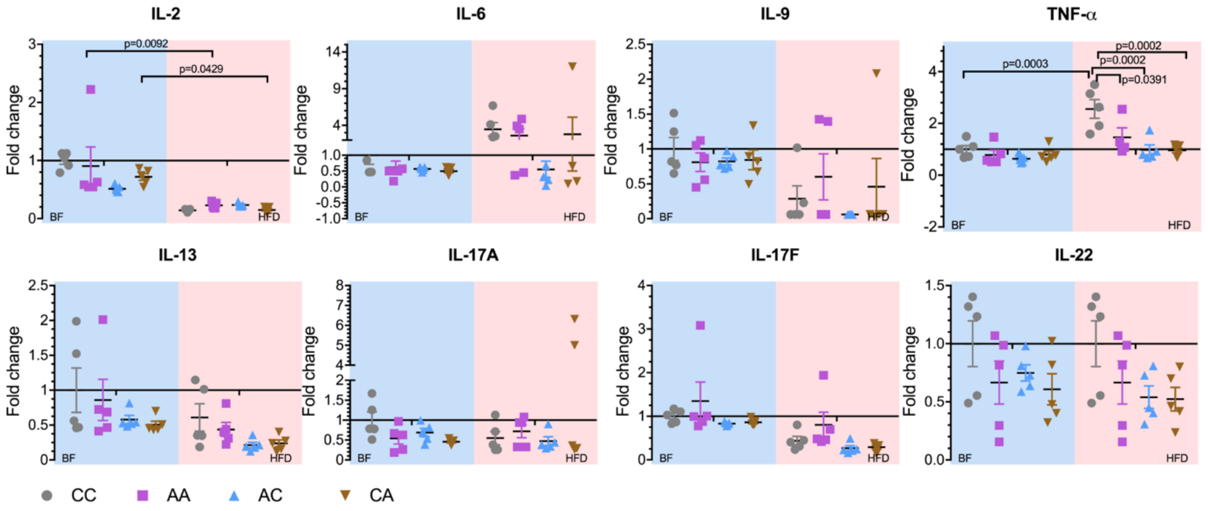

Fig. S4. Mice serum cytokine levels during breastfeeding (BF) and high-fat diet period (HFD). BF samples were collected when pups were 3-weeks old; HFD samples were collected when pups were 22-weeks old. Data represented as mean ± standard error of the mean, two-way ANOVA.

Fig. S5 Uncropped Western Blot images with unadjusted contrast

Figure 4B

Occludin

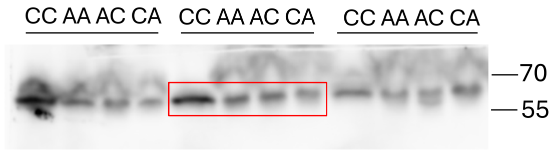

β-actin

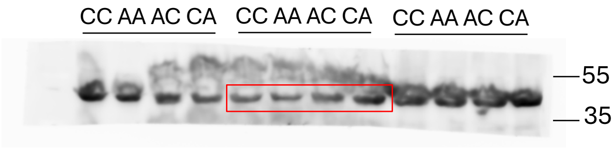

Claudin-1

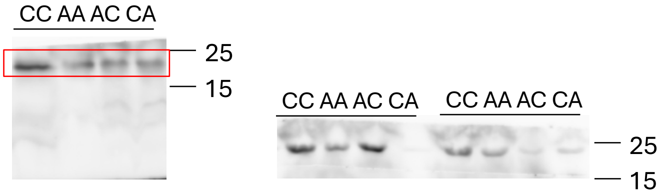

Figure 6B

Upper panel

Occludin

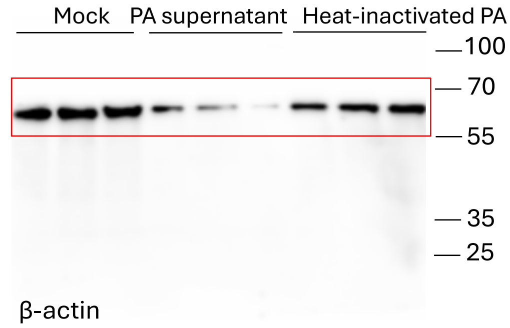

β-actin

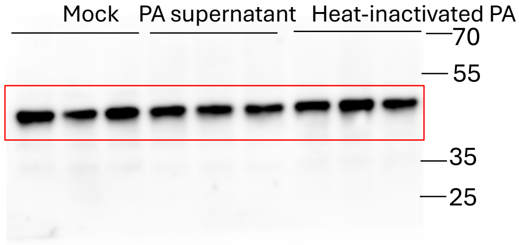

Claudin-1

Mock PA supernatant Heat-inactivated PA

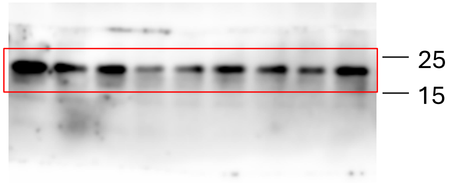

Lower panel

Occludin

Mock LPS_E.coli LPS_PA

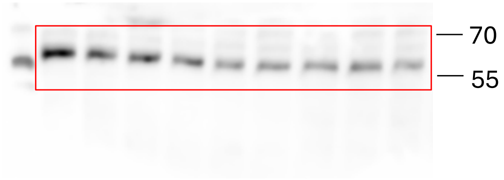

Claudin-1

Mock LPS_E.coli LPS_PA

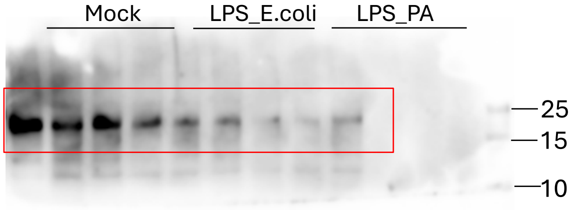

β-actin

Mock LPS_E.coli LPS_PA

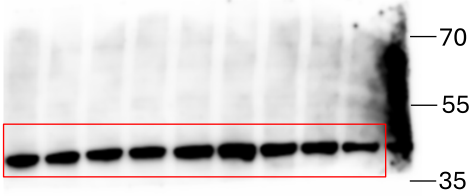

Figure 6J

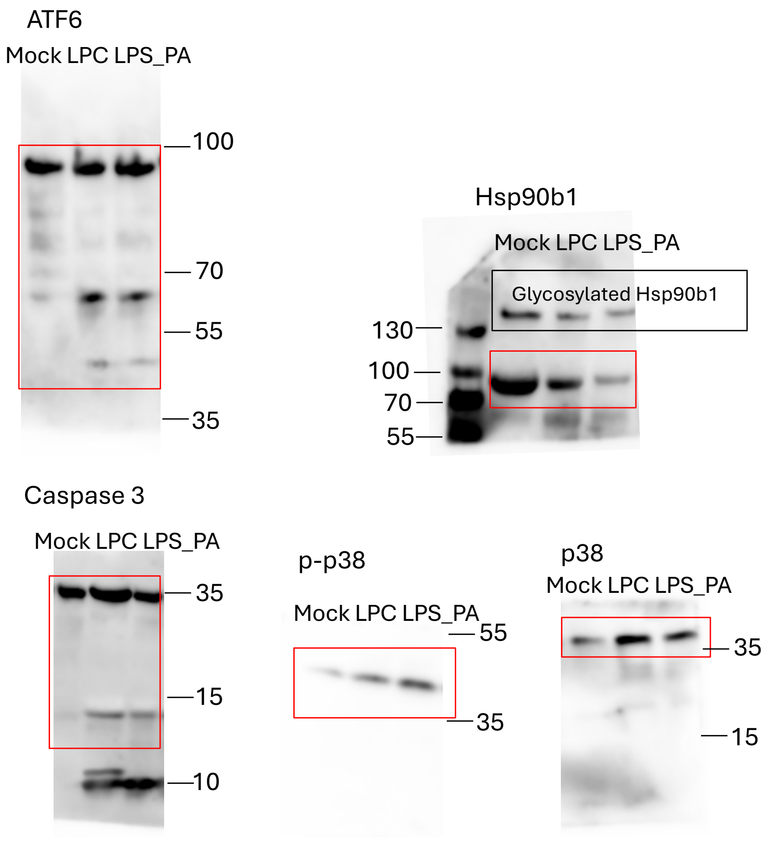

Red box indicates how the blot was cut.

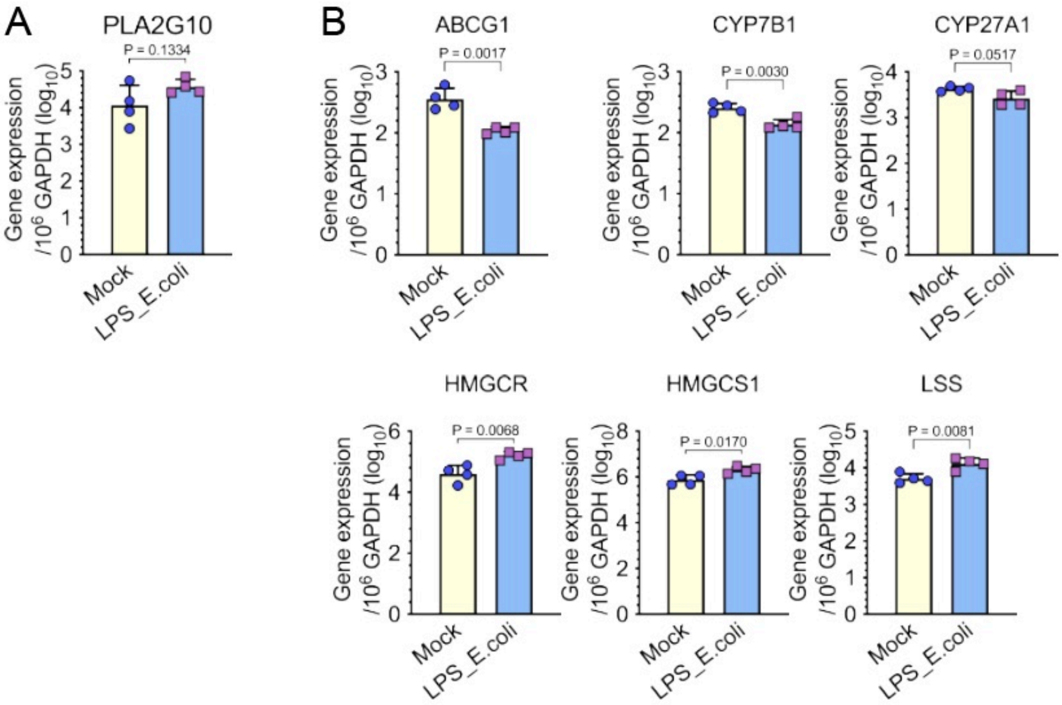

Fig. S6. LPS extract from *E. coli* induced metabolic dysfunctions through the gut-liver axis. The expression of PLA2G10, a gene encoding sPLA2-X in HRT18 cells with or without LPS_E.coli treatment. (H) Expression of gene markers in cholesterol synthesis (HMGCR, HMGCS1, LSS), export (ABCG1) and utilization (CYP7B1, CYP27A1) pathways in Huh7 cells treated with DMSO, LPS_E.coli (50 ng/mL). Data represented as mean ± SEM, n = 4, Student's t-test.

Table S1. Gut microbial taxa in dam-pup dyads

Genus	Phylum	CC dyads	Average taxa abundance		AC dyads	Average taxa abundance		AA dyads	Average taxa abundance		CA of dyads	Average taxa abundance	
			CC dam	CC pup		AC dam	AC pup		AA dam	AA pup		CA dam	CA pup
Acetatifactor_muris	Firmicutes	4	0.4668	0.5802	6	0.4444	1.5317	2	0.5501	0.0656	3	0.4922	1.2811
Acetatifactor	Firmicutes	3	0.0940	0.2878	1	0.0045	0.2252	0	0.0383	0.0000	1	0.0209	0.0418
Alistipes	Bacteroidota	5	1.8136	1.4720	1	0.0343	0.1820	0	0.0360	0.0000	0	0.0000	0.0000
Anaerotruncus	Firmicutes	1	0.0119	0.0268	3	0.0910	0.1954	0	0.0477	0.0000	1	0.0984	0.0522
ASF356	Firmicutes	3	0.2670	0.3400	2	0.0790	0.1745	1	0.1064	0.0388	2	0.0820	0.4012
Bacteroides	Bacteroidota	6	0.6652	2.7218	6	0.7994	15.7733	5	2.9858	26.9635	4	0.7353	21.7837
Blautia	Firmicutes	1	0.0254	0.0328	2	0.1119	0.2028	0	0.0524	0.0000	2	0.1119	0.2983
Butyricicoccus	Firmicutes	5	0.3878	0.3251	5	0.2729	0.3535	0	0.1044	0.0000	3	0.2103	0.7084
Clostridia_UCG-014	Firmicutes	4	0.1074	0.4444	3	0.0597	0.3773	0	0.0728	0.0000	0	0.0000	0.0000
Clostridia_vadinBB60_group	Firmicutes	3	0.0597	0.4042	2	0.0209	0.0895	1	0.0348	0.2908	3	0.1044	0.1939
Enterorhabdus	Actinobacteriota	3	0.3087	0.1148	0	0.5354	0.2401	1	0.3336	0.0567	0	0.0000	0.0000
Helicobacter	Campilobacterota	4	0.1417	0.3281	3	0.0522	0.4176	3	0.1186	0.3997	3	0.0850	0.5086
Incertae_Sedis	Firmicutes	5	0.3937	0.1566	3	0.2177	0.3460	0	0.0940	0.0000	4	0.1566	0.3251
Lachnoclostridium	Firmicutes	5	1.0679	0.6353	4	1.1797	1.3289	4	1.6515	7.5600	5	1.0515	6.9888
Lachnospira	Firmicutes	2	0.1805	0.5324	3	0.1596	0.4012	0	0.0935	0.0000	0	0.0000	0.0000
Lachnospiraceae_FCS020_group	Firmicutes	3	0.2491	0.1909	2	0.2148	0.4996	0	0.1191	0.0000	4	0.3102	1.1126
Lachnospiraceae_NK4A136_group	Firmicutes	3	0.2998	0.3639	3	0.1104	0.1924	0	0.0505	0.0000	1	0.0895	0.2521
Lachnospiraceae_UCG-006	Firmicutes	5	0.4683	0.4415	6	0.6726	0.7114	1	0.2307	0.4191	4	0.4340	0.9724
Lactobacillus	Firmicutes	6	17.1022	25.4571	4	35.6525	5.5526	1	14.0741	0.3550	3	13.0172	9.4541
Muribaculaceae	Bacteroidota	6	6.2774	7.1037	5	3.8986	1.7181	4	3.4387	2.7815	4	4.5891	0.6353
Muribaculum	Bacteroidota	6	13.7584	13.6480	6	6.4265	8.0567	4	8.7912	4.7845	2	3.0395	0.7397
Odoribacter	Bacteroidota	6	1.4497	1.1051	1	0.1253	0.0716	0	0.0328	0.0000	0	0.0000	0.0000
Oscillibacter	Firmicutes	3	0.0955	0.1969	2	0.0701	0.1133	2	0.1305	0.3057	3	0.1969	0.2237
Prevotellaceae_UCG-001	Bacteroidota	4	0.2878	3.4795	1	0.1268	0.0955	0	0.0370	0.0000	0	0.0000	0.0000
Rikenellaceae_RC9_gut_group	Bacteroidota	4	0.2506	0.4713	2	0.1894	0.5309	0	0.1201	0.0000	0	0.0000	0.0000
Sellimonas	Firmicutes	1	0.0358	0.1417	3	0.1268	0.3177	2	0.1934	2.3356	0	0.0000	0.0000
Streptococcus	Firmicutes	4	0.0969	0.1372	0	0.0000	0.0000	0	0.0000	0.0000	6	0.1760	0.2714

CC/AC/AA/CA dyads: the number of dam-pup dyads that shared the respective taxa

Table S2. Lipid-gene interaction network

AA vs CC

Lipid											Gene				Correlation		
Lipid group	Lipid ID	Adduct	Lipid aundance_CC	Lipid aundance_AA	Lipid aundance_AC	Lipid aundance_CA	ratio_AA VS CC	ratio_AC VS CC	ratio_CA VS CC	VIP	Gene ID	Gene name	Gene fold change	p value_gene fold change	Lipid-gene interactions	Spearman's rank correlation coefficiency	p value_correlation
LPC(14:0)	0.91_468.309003	M+H	6812061.999	10512651.83	14233335.7	8901674.605	1.543240774	2.089431321	1.306751848	1.2118173	ENSMUSG00000026663.7	ATF6	0.92373662	0.69047619	12	-0.467012616	0.024659818
LPC(14:0)	0.91_468.309003	M+H	6812061.999	10512651.83	14233335.7	8901674.605	1.543240774	2.089431321	1.306751848	1.2118173	ENSMUSG00000021116.11	EIF2α(EIF2S1)	3.30307383	0.03174603	12	0.436758893	0.038399364
LPC(14:0)	0.91_468.309003	M+H	6812061.999	10512651.83	14233335.7	8901674.605	1.543240774	2.089431321	1.306751848	1.2118173	ENSMUSG00000031077.8	FADD	0.5726326	0.15079365	12	-0.562391912	0.005216828
LPC(14:0)	0.91_468.309003	M+H	6812061.999	10512651.83	14233335.7	8901674.605	1.543240774	2.089431321	1.306751848	1.2118173	ENSMUSG00000020048.14	HSP90B1	4.59748032	0.00793651	12	0.445652174	0.034304023
LPC(14:0)	0.91_468.309003	M+H	6812061.999	10512651.83	14233335.7	8901674.605	1.543240774	2.089431321	1.306751848	1.2118173	ENSMUSG00000004221.17	IKBKG	1.79338853	0.01587302	12	0.340909091	0.111784595
LPC(14:0)	0.91_468.309003	M+H	6812061.999	10512651.83	14233335.7	8901674.605	1.543240774	2.089431321	1.306751848	1.2118173	ENSMUSG00000030265.15	RAS(KRAS)	5.06971122	0.00793651	12	-0.382505571	0.071648117
LPC(14:0)	0.91_468.309003	M+H	6812061.999	10512651.83	14233335.7	8901674.605	1.543240774	2.089431321	1.306751848	1.2118173	ENSMUSG00000020623.12	MKK6(MAP2K6)	0.25553044	0.00793651	12	0.357795909	0.093687955
LPC(14:0)	0.91_468.309003	M+H	6812061.999	10512651.83	14233335.7	8901674.605	1.543240774	2.089431321	1.306751848	1.2118173	ENSMUSG00000020941.8	NIK(MAP3K14)	1.09085444	1	12	-0.458117138	0.027924701
LPC(14:0)	0.91_468.309003	M+H	6812061.999	10512651.83	14233335.7	8901674.605	1.543240774	2.089431321	1.306751848	1.2118173	ENSMUSG00000071369.12	ASK1(MAP3K5)	2.13806677	1	12	-0.568322231	0.004664533
LPC(14:0)	0.91_468.309003	M+H	6812061.999	10512651.83	14233335.7	8901674.605	1.543240774	2.089431321	1.306751848	1.2118173	ENSMUSG00000000440.13	PPARG	2.92849265	0.05555556	12	0.331027668	0.12307867
LPC(14:0)	0.91_468.309003	M+H	6812061.999	10512651.83	14233335.7	8901674.605	1.543240774	2.089431321	1.306751848	1.2118173	ENSMUSG00000024401.15	TNF	0.89526008	0.67233581	12	-0.304347826	0.157744677
LPC(14:0)	0.91_468.309003	M+H	6812061.999	10512651.83	14233335.7	8901674.605	1.543240774	2.089431321	1.306751848	1.2118173	ENSMUSG00000026942.14	TRAF2	0.47524644	0.03174603	12	0.402173913	0.058174596
LPC(17:1)	1.03_552.33064673913	M+HCOO	672647.1093	1008370.819	1027621.153	798257.5649	1.499108232	1.527727004	1.186740497	1.9186668	ENSMUSG00000026663.7	ATF6	0.92373662	0.69047619	13	0.356719368	0.095345146
LPC(17:1)	1.03_552.33064673913	M+HCOO	672647.1093	1008370.819	1027621.153	798257.5649	1.499108232	1.527727004	1.186740497	1.206095	ENSMUSG00000021116.11	EIF2α(EIF2S1)	3.30307383	0.03174603	13	-0.491228085	0.017296473
LPC(17:1)	1.03_552.33064673913	M+HCOO	672647.1093	1008370.819	1027621.153	798257.5649	1.499108232	1.527727004	1.186740497	1.206095	ENSMUSG00000031077.8	FADD	0.5726326	0.15079365	13	-0.414562935	0.04920238
LPC(17:1)	1.03_552.33064673913	M+HCOO	672647.1093	1008370.819	1027621.153	798257.5649	1.499108232	1.527727004	1.186740497	1.9186668	ENSMUSG00000020048.14	HSP90B1	4.59748032	0.00793651	13	0.501976285	0.015777716
LPC(17:1)	1.03_552.33064673913	M+HCOO	672647.1093	1008370.819	1027621.153	798257.5649	1.499108232	1.527727004	1.186740497	1.9953533	ENSMUSG00000030265.15	RAS(KRAS)	5.06971122	0.00793651	13	-0.471343874	0.024406291
LPC(17:1)	1.03_552.33064673913	M+HCOO	672647.1093	1008370.819	1027621.153	798257.5649	1.499108232	1.527727004	1.186740497	2.3923199	ENSMUSG00000004936.9	MEK1(MAP2K1)	1.12243611	0.84126984	13	0.422924901	0.04553753
LPC(17:1)	1.03_552.33064673913	M+HCOO	672647.1093	1008370.819	1027621.153	798257.5649	1.499108232	1.527727004	1.186740497	2.3923199	ENSMUSG00000018932.10	MKK3(MAP2K3)	0.52906915	0.01587302	13	0.330039526	0.124252687
LPC(17:1)	1.03_552.33064673913	M+HCOO	672647.1093	1008370.819	1027621.153	798257.5649	1.499108232	1.527727004	1.186740497	2.3923199	ENSMUSG00000020623.12	MKK6(MAP2K6)	0.25553044	0.00793651	13	-0.508524849	0.013220356
LPC(17:1)	1.03_552.33064673913	M+HCOO	672647.1093	1008370.819	1027621.153	798257.5649	1.499108232	1.527727004	1.186740497	1.9953533	ENSMUSG00000020366.19	JNK2(MAPK9)	0.93099621	1	13	-0.508524849	0.013220356
LPC(17:1)	1.03_552.33064673913	M+HCOO	672647.1093	1008370.819	1027621.153	798257.5649	1.499108232	1.527727004	1.186740497	1.9953533	ENSMUSG00000000440.13	PPARG	2.92849265	0.05555556	13	0.330039526	0.124252687
LPC(17:1)	1.03_552.33064673913	M+HCOO	672647.1093	1008370.819	1027621.153	798257.5649	1.499108232	1.527727004	1.186740497	2.3923199	ENSMUSG00000031887.14	TRADD	0.47964951	0.05555556	13	-0.471343874	0.024406291
LPC(17:1)	1.03_552.33064673913	M+HCOO	672647.1093	1008370.819	1027621.153	798257.5649	1.499108232	1.527727004	1.186740497	1.9186668	ENSMUSG00000026942.14	TRAF2	1.47524644	1.03174603	13	-0.361255262	0.090331888
LPC(17:1)	1.03_552.33064673913	M+HCOO	672647.1093	1008370.819	1027621.153	798257.5649	1.499108232	1.527727004	1.186740497	1.9953533	ENSMUSG00000020484.20	XBP1	0.71865981	0.15079365	13	0.422924901	0.04553753
LPC(20:2)	1.03_552.33064673913	M+HCOO	1575005.581	2159655.411	2038617.545	1652422.984	1.371204926	1.294355759	1.049153732	1.3060769	ENSMUSG00000022136.9	HSP40(DNAJC3)	2.65388089	0.00793651	16	0.337944664	0.115088472
LPC(20:2)	1.03_552.33064673913	M+HCOO	1575005.581	2159655.411	2038617.545	1652422.984	1.371204926	1.294355759	1.049153732	1.3060769	ENSMUSG00000021116.11	EIF2α(EIF2S1)	3.30307383	0.03174603	16	0.517786561	0.012421897
LPC(20:2)	0.91_468.309003	M+HCOO	1575005.581	2159655.411	2038617.545	1652422.984	1.371204926	1.294355759	1.049153732	1.2118173	ENSMUSG00000031077.8	FADD	0.5726326	0.15079365	16	-0.39229249	0.065084096
LPC(20:2)	1.03_552.33064673913	M+HCOO	1575005.581	2159655.411	2038617.545	1652422.984	1.371204926	1.294355759	1.049153732	1.3060769	ENSMUSG00000020048.14	HSP90B1	4.59748032	0.00793651	16	0.301383399	0.161988236
LPC(20:2)	1.23_548.37257786956	M+HCOO	1575005.581	2159655.411	2038617.545	1652422.984	1.371204926	1.294355759	1.049153732	1.7568834	ENSMUSG00000052684.5	JUN	3.72861127	0.00793651	16	0.427865613	0.042876506
LPC(20:2)	1.24_592.36229652173	M+HCOO	1575005.581	2159655.411	2038617.545	1652422.984	1.371204926	1.294355759	1.049153732	1.1606002	ENSMUSG00000030265.15	RAS(KRAS)	5.06971122	0.00793651	16	-0.317193676	0.140273699
LPC(20:2)	1.23_548.37257786956	M+HCOO	1575005.581	2159655.411	2038617.545	1652422.984	1.371204926	1.294355759	1.049153732	1.7568834	ENSMUSG00000035027.19	MEK1(MAP2K2)	0.9468945	0.69047619	16	0.308300395	0.152211162
LPC(20:2)	1.23_548.37257786956	M+HCOO	1575005.581	2159655.411	2038617.545	1652422.984	1.371204926	1.294355759	1.049153732	1.7568834	ENSMUSG00000018932.10	MKK3(MAP2K3)	0.52906915	0.01587302	16	-0.396245059	0.062247604
LPC(20:2)	0.93_588.33103530434	M+HCOO	1575005.581	2159655.411	2038617.545	1652422.984	1.371204926	1.294355759	1.049153732	1.8897137	ENSMUSG00000020623.12	MKK6(MAP2K6)	0.25553044	0.00793651	16	0.31916996	0.137715996
LPC(20:2)	1.23_548.37257786956	M+HCOO	1575005.581	2159655.411	2038617.545	1652422.984	1.371204926	1.294355759	1.049153732	1.7568834	ENSMUSG00000002948.20	MKK7(MAP2K7)	0.29104921	0.01587302	16	-0.368577075	0.084266788
LPC(20:2)	1.23_548.37257786956	M+HCOO	1575005.581	2159655.411	2038617.545	1652422.984	1.371204926	1.294355759	1.049153732	1.7568834	ENSMUSG00000020366.19	JNK2(MAPK10)	1.93099621	1	16	0.431818182	0.040838014
LPC(20:2)	1.24_592.36229652173	M+HCOO	1575005.581	2159655.411	2038617.545	1652422.984	1.371204926	1.294355759	1.049153732	1.1606002	ENSMUSG00000028698.14	PI3K-C2γ(PIK3R3)	1.656932	0.69047619	16	0.480237154	0.021578917
LPC(20:2)	1.24_592.36229652173	M+HCOO	1575005.581	2159655.411	2038617.545	1652422.984	1.371204926	1.294355759	1.049153732	1.1606002	ENSMUSG00000000440.13	PPARG	2.92849265	0.05555556	16	0.448616601	0.033019703
LPC(20:2)	0.93_588.33103530434	M+HCOO	1575005.581	2159655.411	2038617.545	1652422.984	1.371204926	1.294355759	1.049153732	1.8897137	ENSMUSG00000031887.14	TRADD	0.47964951	0.05555556	16	0.311264822	0.148153613
LPC(20:2)	1.03_552.33064673913	M+HCOO	1575005.581	2159655.411	2038617.545	1652422.984	1.371204926	1.294355759	1.049153732	1.3060769	ENSMUSG00000026942.14	TRAF2	2.47524644	2.03174603	16	-0.461462451	0.027893837
LPC(20:2)	1.03_552.33064673913	M+HCOO	1575005.581	2159655.411	2038617.545	1652422.984	1.371204926	1.294355759	1.049153732	1.3060769	ENSMUSG00000020484.20	XBP1	0.71865981	0.15079365	16	-0.481225296	2.13E-02
LPC(20:2)	0.94_544.34120230434	M+H	7025246.705	12492375.77	13536831.19	8649471.031	1.778211683	1.92688339	1.23119819	1.944648	ENSMUSG00000022136.9	HSP40(DNAJC3)	2.65388089	0.00793651	16	0.374104286	0.078647267
LPC(20:2)	0.93_588.33103530434	M+H	7025246.705	12492375.77	13536831.19	8649471.031	1.778211683	1.92688339	1.23119819	1.8897137	ENSMUSG00000021116.11	EIF2α(EIF2S1)	3.30307383	0.03174603	16	-0.385375494	0.070288548
LPC(20:2)	0.93_588.33103530434	M+H	7025246.705	12492375.77	13536831.19	8649471.031	1.778211683	1.92688339	1.23119819	1.8897137	ENSMUSG00000031077.8	FADD	0.5726326	0.15079365	16	-0.301383399	0.161988236
LPC(20:2)	0.93_588.33103530434	M+H	7025246.705	12492375.77	13536831.19	8649471.031	1.778211683	1.92688339	1.23119819	1.8897137	ENSMUSG00000020048.14	HSP90B1	4.59748032	0.00793651	16	-0.303359684	0.15915026
LPC(20:2)	3.07_580.43396160869	M+H	7025246.705	12492375.77	13536831.19	8649471.031	1.778211683	1.92688339	1.23119819	1.206095	ENSMUSG00000030265.15	RAS(KRAS)	5.06971122	0.00793651	16	0.308300395	0.152211162
LPC(20:2)	1.14_572.37172873913	M+H	7025246.705	12492375.77	13536831.19	8649471.031	1.778211683	1.92688339	1.23119819	1.9186668	ENSMUSG00000004936.9	MEK2(MAP2K2)	1.12243611	0.84126984	16	0.502964427	0.015548148
LPC(20:2)	0.89_612.33280752173	M+H	7025246.705	12492375.77	13536831.19	8649471.031	1.778211683	1.92688339	1.23119819	1.9953533	ENSMUSG00000020623.12	MKK6(MAP2K6)	0.25553044	0.00793651	16	0.476284585	0.022800557
LPC(20:2)	1.14_572.37172873913	M+H	7025246.705	12492375.77	13536831.19	8649471.031	1.778211683	1.92688339	1.23119819	1.9186668	ENSMUSG00000002948.20	MKK7(MAP2K7)	0.29104921	0.01587302	16	-0.504940711	0.015097266
LPC(20:2)	3.07_580.43396160869	M+H	7025246.705	12492375.77	13536831.19	8649471.031	1.778211683	1.92688339	1.23119819	1.206095	ENSMUSG00000053436.16	p38γ (MAPK12)	1.10675829	0.67517362	16	-0.442687747	0.035628166
LPC(20:2)	3.07_580.43396160869	M+H	7025246.705	12492375.77	13536831.19	8649471.031	1.778211683	1.92688339	1.23119819	1.206095	ENSMUSG00000020366.19	JNK2(MAPK11)	2.93099621	1	16	-0.316205534	0.141565411
LPC(20:2)	3.07_580.43396160869	M+H	7025246.705	12492375.77	13536831.19	8649471.031	1.778211683	1.92688339	1.23119819	1.206095	ENSMUSG00000028698.14	PI3K-C2γ(PIK3R4)	2.656932	1.69047619	16	0.351778656	0.100272723
LPC(20:2)	3.07_580.43396160869	M+H	7025246.705	12492375.77	13536831.19	8649471.031	1.778211683	1.92688339	1.23119819	1.206095	ENSMUSG00000000440.13	PPARG	2.92849265	0.05555556	16	0.345935271	0.10589902
LPC(20:2)	1.14_572.37172873913	M+H	7025246.705	12492375.77	13536831.19	8649471.031	1.778211683	1.92688339	1.23119819	1.9186668	ENSMUSG00000000441.18	RAF1	1.138275	0.69047619	16	0.462450593	0.027527897
LPC(20:2)	0.89_612.33280752173	M+H	7025246.705	12492375.77	13536831.19	8649471.031	1.778211683	1.92688339	1.23119819	1.9953533	ENSMUSG00000031887.14	TRADD	0.47964951	0.05555556	16	0.540513834	0.00865021
LPC(20:2)	0.94_544.34120230434	M+H	7025246.705	12492375.77	13536831.19	8649471.031	1.778211683	1.92688339	1.23119819	1.944648	ENSMUSG00000026942.14	TRAF2	3.47524644	3.03174603	16	-0.385375494	0.070288548
LPC(20:2)	3.07_580.43396160869	M+H	7025246.705	12492375.77	13536831.19	8649471.031	1.778211683	1.92688339	1.23119819	1.206095	ENSMUSG00000020484.20	XBP1	0.71865981	0.15079365	16	0.539525692	0.008791485
LPC(20:4)	0.89_612.33280752173	M+HCOO	150822398.6	267574750.1	230639562.1	219933215.7	1.774104858	1.52921293	1.458226482	1.9953533	ENSMUSG00000026029.15	CASP8	0.48775428	0.03174603	13	-0.377470356	0.076624247
LPC(20:4)	0.89_612.33280752173	M+HCOO	150822398.6	267574750.1	230639562.1	219933215.7	1.774104858	1.52921293	1.458226482	1.9953533	ENSMUSG00000021116.11	EIF2α(EIF2S1)	3.30307383	0.03174603	13	-0.491106719	0.018492407
LPC(20:4)	0.89_612.33280752173	M+HCOO	150822398.6	267574750.1	230639562.1	219933215.7	1.774104858	1.52921293	1.458226482	1.9953533	ENSMUSG00000031077.8	FADD	0.5726326	0.15079365	13	-0.419960474	0.047195951
LPC(20:4)	1.24_592.36229652173	M+HCOO	150822398.6	267574750.1	230639562.1	219933215.7	1.774104858	1.52921293	1.458226482	1.1606002	ENSMUSG00000004221.17	IKBKG	1.79338853	0.01587302	13	0.353754941	0.098279217
LPC(20:4)	0.89_612.33280752173	M+HCOO	150822398.6	267574750.1	230639562.1	219933215.7	1.774104858	1.52921293	1.458226482	2.3923199	ENSMUSG00000030265.15	RAS(KRAS)	5.06971122	0.00793651	13	-0.491106719	0.018492407
LPC(20:4)	0.94_544.34120230434	M+HCOO	150822398.6	267574750.1	230639562.1	219933215.7	1.774104858	1.52921293	1.458226482	1.944648	ENSMUSG00000020623.12	MKK6(MAP2K6)	0.25553044	0.00793651	13	0.392834862	0.063702653

LPC(20:4)	0.93_588.331035304348	M+HCOO	150822398.6	267574750.1	230639562.1	219933215.7	1.774104858	1.52921293	1.458226482	1.8897137	ENSMUSG00000020941.8	NIK(MAP3K14)	1.09085444	1	13	0.300395257	0.163420675
LPC(20:4)	1.23_548.372577869565	M+HCOO	150822398.6	267574750.1	230639562.1	219933215.7	1.774104858	1.52921293	1.458226482	1.7568834	ENSMUSG00000071369.12	ASK1(MAP3K6)	2.13806677	1	13	0.330971104	0.122924292
LPC(20:4)	1.24_592.362296521739	M+HCOO	150822398.6	267574750.1	230639562.1	219933215.7	1.774104858	1.52921293	1.458226482	1.1606002	ENSMUSG00000053436.16	p38α(MAPK14)	1.97725607	0.15079365	13	0.315505165	0.142514994
LPC(20:4)	0.89_612.332807521739	M+HCOO	150822398.6	267574750.1	230639562.1	219933215.7	1.774104858	1.52921293	1.458226482	2.3923199	ENSMUSG00000028698.14	PI3K-C2γ(PIK3R5)	3.656932	2.69047619	13	-0.377470356	0.076624247
LPC(20:4)	0.89_612.332807521739	M+HCOO	150822398.6	267574750.1	230639562.1	219933215.7	1.774104858	1.52921293	1.458226482	2.3923199	ENSMUSG00000000440.13	PPARG	2.92849265	0.05555556	13	-0.483201581	0.020698212
LPC(20:4)	0.89_612.332807521739	M+HCOO	150822398.6	267574750.1	230639562.1	219933215.7	1.774104858	1.52921293	1.458226482	2.3923199	ENSMUSG00000040435.13	GADD34(PPP1R15A)	0.50828146	0.05555556	13	0.468379447	0.025413081
LPC(20:4)	0.89_612.332807521739	M+HCOO	150822398.6	267574750.1	230639562.1	219933215.7	1.774104858	1.52921293	1.458226482	2.3923199	ENSMUSG00000026942.14	TRAF2	4.47524644	4.03174603	13	0.513833992	0.013199547
LPC(20:4)	0.89_612.332807521739	M+HCOO	150822398.6	267574750.1	230639562.1	219933215.7	1.774104858	1.52921293	1.458226482	2.3923199	ENSMUSG00000020484.20	XBP1	0.71865981	0.15079365	13	0.476284585	0.022800557
LPC(20:4)	1.24_592.362296521739	M+H	1025081106	1946614008	1742498230	1503639564	1.898985355	1.699863767	1.46684936	1.1606002	ENSMUSG00000022136.9	HSP40(DNAJC3)	2.65388089	0.00793651	11	0.468495196	0.024146747
LPC(20:4)	0.91_468.309003	M+H	1025081106	1946614008	1742498230	1503639564	1.898985355	1.699863767	1.46684936	1.2118173	ENSMUSG00000031077.8	FADD	0.5726326	0.15079365	11	0.442302954	0.034573597
LPC(20:4)	0.91_468.309003	M+H	1025081106	1946614008	1742498230	1503639564	1.898985355	1.699863767	1.46684936	1.2118173	ENSMUSG00000004221.17	IKBKG	1.79338853	0.01587302	11	0.382411067	0.072615126
LPC(20:4)	0.89_612.332807521739	M+H	1025081106	1946614008	1742498230	1503639564	1.898985355	1.699863767	1.46684936	1.9953533	ENSMUSG00000030265.15	RAS(KRAS)	5.06971122	0.00793651	11	0.481838414	0.019903993
LPC(20:4)	0.91_468.309003	M+H	1025081106	1946614008	1742498230	1503639564	1.898985355	1.699863767	1.46684936	1.2118173	ENSMUSG00000020623.12	MKK6(MAP2K6)	0.25553044	0.00793651	11	-0.323225605	0.132475856
LPC(20:4)	0.91_468.309003	M+H	1025081106	1946614008	1742498230	1503639564	1.898985355	1.699863767	1.46684936	1.2118173	ENSMUSG00000020941.8	NIK(MAP3K14)	1.09085444	1	11	-0.353138951	0.098350992
LPC(20:4)	0.89_612.332807521739	M+H	1025081106	1946614008	1742498230	1503639564	1.898985355	1.699863767	1.46684936	2.3923199	ENSMUSG00000053436.16	p38α(MAPK14)	1.97725607	0.15079365	11	0.481838414	0.019903993
LPC(20:4)	0.93_588.331035304348	M+H	1025081106	1946614008	1742498230	1503639564	1.898985355	1.699863767	1.46684936	1.8897137	ENSMUSG00000028698.14	PI3K-C2γ(PIK3R6)	4.656932	3.69047619	11	0.330615281	0.123351845
LPC(20:4)	1.14_572.37172873913	M+H	1025081106	1946614008	1742498230	1503639564	1.898985355	1.699863767	1.46684936	1.9186668	ENSMUSG00000000440.13	PPARG	2.92849265	0.05555556	11	0.525327419	0.010049065
LPC(20:4)	1.23_548.372577869565	M+H	1025081106	1946614008	1742498230	1503639564	1.898985355	1.699863767	1.46684936	1.7568834	ENSMUSG00000040435.13	GADD34(PPP1R15A)	0.50828146	0.05555556	11	0.40672104	0.054104983
LPC(20:4)	0.91_468.309003	M+H	1025081106	1946614008	1742498230	1503639564	1.898985355	1.699863767	1.46684936	1.2118173	ENSMUSG00000000441.18	RAF1	1.138275	0.69047619	11	0.37944664	0.075000674
LPC(22:0)	1.03_552.33064673913	M+H	1939988.712	2498346.356	2420156.701	2389134.987	1.287814893	1.247510713	1.231520046	1.3060769	ENSMUSG00000026663.7	ATF6	0.92373662	0.69047619	22	-0.369565217	0.083389986
LPC(22:0)	1.03_552.33064673913	M+H	1939988.712	2498346.356	2420156.701	2389134.987	1.287814893	1.247510713	1.231520046	1.3060769	ENSMUSG00000031628.10	CASP3	1.80010753	0.24915264	22	0.516798419	0.012612657
LPC(22:0)	1.03_552.33064673913	M+H	1939988.712	2498346.356	2420156.701	2389134.987	1.287814893	1.247510713	1.231520046	1.3060769	ENSMUSG00000025076.13	CASP7	1.3699966	0.54761905	22	0.359683794	0.092477736
LPC(22:0)	0.91_468.309003	M+H	1939988.712	2498346.356	2420156.701	2389134.987	1.287814893	1.247510713	1.231520046	1.2118173	ENSMUSG00000028914.14	CASP9	0.94119011	1	22	-0.536561265	0.009226897
LPC(22:0)	1.24_592.362296521739	M+H	1939988.712	2498346.356	2420156.701	2389134.987	1.287814893	1.247510713	1.231520046	1.1606002	ENSMUSG00000020715.10	IRE1α(ERN1)	0.95601339	1	22	0.527667984	0.010642279
LPC(22:0)	1.03_552.33064673913	M+H	1939988.712	2498346.356	2420156.701	2389134.987	1.287814893	1.247510713	1.231520046	1.3060769	ENSMUSG00000031077.8	FADD	0.5726326	0.15079365	22	-0.361660079	0.090602737
LPC(22:0)	1.03_552.33064673913	M+H	1939988.712	2498346.356	2420156.701	2389134.987	1.287814893	1.247510713	1.231520046	1.3060769	ENSMUSG00000020048.14	HSP90B1	4.59748032	0.00793651	22	-0.384387352	0.071057578
LPC(22:0)	1.24_592.362296521739	M+H	1939988.712	2498346.356	2420156.701	2389134.987	1.287814893	1.247510713	1.231520046	1.1606002	ENSMUSG00000030265.15	RAS(KRAS)	5.06971122	0.00793651	22	-0.32312253	0.132702731
LPC(22:0)	1.24_592.362296521739	M+H	1939988.712	2498346.356	2420156.701	2389134.987	1.287814893	1.247510713	1.231520046	1.1606002	ENSMUSG00000018932.10	MKK3(MAP2K3)	0.52906915	0.01587302	22	-0.408102767	0.054313174
LPC(22:0)	1.23_548.372577869565	M+H	1939988.712	2498346.356	2420156.701	2389134.987	1.287814893	1.247510713	1.231520046	1.7568834	ENSMUSG00000020623.12	MKK6(MAP2K6)	0.25553044	0.00793651	22	0.389328063	0.067276608
LPC(22:0)	1.24_592.362296521739	M+H	1939988.712	2498346.356	2420156.701	2389134.987	1.287814893	1.247510713	1.231520046	1.1606002	ENSMUSG00000022610.11	p38γ (MAPK12)	1.10675829	0.67517362	22	-0.301383399	0.161988236
LPC(22:0)	1.23_548.372577869565	M+H	1939988.712	2498346.356	2420156.701	2389134.987	1.287814893	1.247510713	1.231520046	1.7568834	ENSMUSG00000004864.14	p38α(MAPK14)	1.06195772	1	22	0.492094862	0.018230788
LPC(22:0)	1.24_592.362296521739	M+H	1939988.712	2498346.356	2420156.701	2389134.987	1.287814893	1.247510713	1.231520046	1.1606002	ENSMUSG00000053436.16	p38α(MAPK14)	1.97725607	0.15079365	22	0.333992095	0.119605691
LPC(22:0)	1.24_592.362296521739	M+H	1939988.712	2498346.356	2420156.701	2389134.987	1.287814893	1.247510713	1.231520046	1.1606002	ENSMUSG00000021936.15	JNK1(MAPK8)	1.84696108	0.22222222	22	-0.305335968	0.156347999
LPC(22:0)	1.24_592.362296521739	M+H	1939988.712	2498346.356	2420156.701	2389134.987	1.287814893	1.247510713	1.231520046	1.1606002	ENSMUSG00000040435.13	GADD34(PPP1R15A)	0.50828146	0.05555556	22	0.366600791	0.086041479
LPC(22:0)	1.24_592.362296521739	M+H	1939988.712	2498346.356	2420156.701	2389134.987	1.287814893	1.247510713	1.231520046	1.1606002	ENSMUSG00000024401.15	TNF	0.89526008	0.67233581	22	-0.349802372	0.102296489
LPC(22:0)	1.24_592.362296521739	M+H	1939988.712	2498346.356	2420156.701	2389134.987	1.287814893	1.247510713	1.231520046	1.1606002	ENSMUSG00000026942.14	TRAF2	5.47524644	5.03174603	22	0.363636364	0.088756722
LPC(22:0)	1.24_592.362296521739	M+H	1939988.712	2498346.356	2420156.701	2389134.987	1.287814893	1.247510713	1.231520046	1.1606002	ENSMUSG00000029380.12	CXCL1	0.14494732	0.01115943	22	-0.638538159	0.00104147
LPC(22:0)	1.24_592.362296521739	M+H	1939988.712	2498346.356	2420156.701	2389134.987	1.287814893	1.247510713	1.231520046	1.1606002	ENSMUSG00000027398.14	IL1β(IL1B)	0.28185479	0.03174603	22	-0.529644269	0.01031311
LPC(22:0)	1.24_592.362296521739	M+H	1939988.712	2498346.356	2420156.701	2389134.987	1.287814893	1.247510713	1.231520046	1.1606002	ENSMUSG00000020048.14	HSP90B1	4.59748032	0.00793651	22	0.47173913	0.047035573
LPC(22:0)	1.24_592.362296521739	M+H	1939988.712	2498346.356	2420156.701	2389134.987	1.287814893	1.247510713	1.231520046	1.1606002	ENSMUSG00000022136.9	HSP40(DNAJC3)	2.65388089	0.00793651	22	0.551778656	0.030272723
LPC(22:0)	1.24_592.362296521739	M+H	1939988.712	2498346.356	2420156.701	2389134.987	1.287814893	1.247510713	1.231520046	1.1606002	ENSMUSG00000026864.14	GRP78	1.2265347	0.30952381	22	0.617391304	0.017522408
LPC(22:4)	1.23_548.372577869565	M+H	7453348.495	15911498.64	9803237.161	12051707.99	2.134812112	1.315279591	1.616952166	1.7568834	ENSMUSG00000022136.9	HSP40(DNAJC3)	2.65388089	0.00793651	16	-0.513833992	0.013199547
LPC(22:4)	1.23_548.372577869565	M+H	7453348.495	15911498.64	9803237.161	12051707.99	2.134812112	1.315279591	1.616952166	1.7568834	ENSMUSG00000021116.11	EIF2α(EIF2S1)	3.30307383	0.03174603	16	-0.389741674	0.066007645
LPC(22:4)	1.23_548.372577869565	M+H	7453348.495	15911498.64	9803237.161	12051707.99	2.134812112	1.315279591	1.616952166	1.7568834	ENSMUSG00000031077.8	FADD	0.5726326	0.15079365	16	-0.380434783	0.074198883
LPC(22:4)	1.23_548.372577869565	M+H	7453348.495	15911498.64	9803237.161	12051707.99	2.134812112	1.315279591	1.616952166	1.7568834	ENSMUSG00000020048.14	HSP90B1	4.59748032	0.00793651	16	-0.580039526	0.004356377
LPC(22:4)	0.93_588.331035304348	M+H	7453348.495	15911498.64	9803237.161	12051707.99	2.134812112	1.315279591	1.616952166	1.8897137	ENSMUSG00000004221.17	IKBKG	1.79338853	0.01587302	16	-0.434782609	0.039360457
LPC(22:4)	0.93_588.331035304348	M+H	7453348.495	15911498.64	9803237.161	12051707.99	2.134812112	1.315279591	1.616952166	1.8897137	ENSMUSG00000052684.5	JUN	3.72861127	0.00793651	16	-0.376482213	0.077446096
LPC(22:4)	0.93_588.331035304348	M+H	7453348.495	15911498.64	9803237.161	12051707.99	2.134812112	1.315279591	1.616952166	1.8897137	ENSMUSG00000030265.15	RAS(KRAS)	5.06971122	0.00793651	16	0.338522373	0.114101774
LPC(22:4)	0.94_544.341202304348	M+H	7453348.495	15911498.64	9803237.161	12051707.99	2.134812112	1.315279591	1.616952166	1.944648	ENSMUSG00000035027.19	MEK1(MAP2K3)	1.9468945	1.69047619	16	0.317193676	0.140273699
LPC(22:4)	0.94_544.341202304348	M+H	7453348.495	15911498.64	9803237.161	12051707.99	2.134812112	1.315279591	1.616952166	1.944648	ENSMUSG00000020623.12	MKK6(MAP2K6)	0.25553044	0.00793651	16	-0.37055336	0.082520168
LPC(22:4)	0.94_544.341202304348	M+H	7453348.495	15911498.64	9803237.161	12051707.99	2.134812112	1.315279591	1.616952166	1.944648	ENSMUSG00000002948.20	MKK7(MAP2K7)	0.29104921	0.01587302	16	-0.336956522	0.116205712
LPC(22:4)	0.93_588.331035304348	M+H	7453348.495	15911498.64	9803237.161	12051707.99	2.134812112	1.315279591	1.616952166	1.8897137	ENSMUSG00000020366.19	JNK2(MAPK12)	3.93099621	1	16	-0.441294806	0.035036525
LPC(22:4)	0.93_588.331035304348	M+H	7453348.495	15911498.64	9803237.161	12051707.99	2.134812112	1.315279591	1.616952166	1.8897137	ENSMUSG00000028698.14	PI3K-C2γ(PIK3R7)	5.656932	4.69047619	16	0.445652174	0.034304023
LPC(22:4)	0.93_588.331035304348	M+H	7453348.495	15911498.64	9803237.161	12051707.99	2.134812112	1.315279591	1.616952166	1.8897137	ENSMUSG00000000440.13	PPARG	2.92849265	0.05555556	16	0.364624506	0.087844503
LPC(22:4)	0.94_544.341202304348	M+H	7453348.495	15911498.64	9803237.161	12051707.99	2.134812112	1.315279591	1.616952166	1.944648	ENSMUSG00000031887.14	TRADD	0.47964951	0.05555556	16	-0.412055336	0.051852839
LPC(22:4)	0.94_544.341202304348	M+H	7453348.495	15911498.64	9803237.161	12051707.99	2.134812112	1.315279591	1.616952166	1.944648	ENSMUSG00000059552.14	p53(TRP53)	0.98704298	0.84126984	16	0.368577075	0.084266788
LPC(22:4)	0.93_588.331035304348	M+H	7453348.495	15911498.64	9803237.161	12051707.99	2.134812112	1.315279591	1.616952166	1.8897137	ENSMUSG00000020484.20	XBP1	0.71865981	0.15079365	16	0.473320158	0.023753305
LPC(22:6)	1.14_572.37172873913	M+HCOO	108946895.9	232028470.6	168236816.7	138776077.3	2.12973916	1.544209363	1.273795606	1.9186668	ENSMUSG00000042406.9	ATF4	0.60874006	0.0952381	34	-0.333992095	0.119605691
LPC(22:6)	0.94_544.341202304348	M+HCOO	108946895.9	232028470.6	168236816.7	138776077.3	2.12973916	1.544209363	1.273795606	1.944648	ENSMUSG00000026029.15	CASP8	0.48775428	0.03174603	34	-0.668972332	0.000662
LPC(22:6)	3.07_580.433961608696	M+HCOO	108946895.9	232028470.6	168236816.7	138776077.3	2.12973916	1.544209363	1.273795606	1.206095	ENSMUSG00000026029.15	CASP8	0.48775428	0.03174603	34	0.396245059	0.062247604
LPC(22:6)	3.07_580.433961608696	M+HCOO	108946895.9	232028470.6	168236816.7	138776077.3	2.12973916	1.544209363	1.273795606	1.206095	ENSMUSG00000022136.9	HSP40(DNAJC3)	2.65388089	0.00793651	34	-0.335968379	0.117330976
LPC(22:6)	1.14_572.37172873913	M+HCOO	108946895.9	232028470.6	168236816.7	138776077.3	2.12973916	1.544209363	1.273795606	1.9186668	ENSMUSG00000022136.9	HSP40(DNAJC3)	2.65388089	0.00793651	34	0.442687747	0.035628166
LPC(22:6)	3.07_580.433961608696	M+HCOO	108946895.9	232028470.6	168236816.7	138776077.3	2.12973916	1.544209363	1.273795606	1.206095	ENSMUSG00000021116.11	EIF2α(EIF2S1)	3.30307383	0.03174603	34	-0.360080351	0.091461514
LPC(22:6)	1.14_572.37172873913	M+HCOO	108946895.9	232028470.6	168236816.7	138776077.3	2.12973916	1.544209363	1.273795606	1.9186668	ENSMUSG00000021116.11	EIF2α(EIF2S1)	3.30307383	0.03174603	34	0.366600791	0.086041479
LPC(22:6)	3.07_580.433961608696	M+HCOO	108946895.9	232028470.6	168236816.7	138776077.3	2.12973916	1.544209363	1.273795606	1.206095	ENSMUSG00000031077.8	FADD	0.5726326	0.15079365	34	0.52173913	0.011682581
LPC(22:6)	3.07_580.433961608696	M+HCOO	108946895.9	232028470.6	168236816.7	138776077.3	2.12973916	1.544209363	1.273795606	1.206095	ENSMUSG00000031077.8	FADD	0.5726326	0.15079365	34	-0.320419828	0.136063786
LPC(22:6)	3.07_580.433961608696	M+HCOO	108946895.9	232028470.6	168236816.7	138776077.3	2.12973916	1.544209363	1.273795606	1.206095	ENSMUSG00000020048.14	HSP90B1	4.59748032	0.00793651	34	-0.342885375	0.109621579
LPC(22:6)	1.14_572.37172873913	M+HCOO	108946895.9	232028470.6	168236816.7	138776077.3	2.12973916	1.544209363	1.273795606	1.9186668	ENSMUSG00000020048.14	HSP90B1	4.59748032	0.00793651	34	0.389328063	0.067276608

LPC(22:6)	0.89_612.33280752173	M+HCOO	108946895.9	232028470.6	168236816.7	138776077.3	2.12973916	1.544209363	1.273795606	1.9953533	ENSMUSG00000004221.17	IKBKG	1.79338853	0.01587302	34	0.342885375	0.109621579
LPC(22:6)	0.89_612.33280752173	M+HCOO	108946895.9	232028470.6	168236816.7	138776077.3	2.12973916	1.544209363	1.273795606	2.3923199	ENSMUSG00000004221.17	IKBKG	1.79338853	0.01587302	34	0.342885375	0.109621579
LPC(22:6)	0.89_612.33280752173	M+HCOO	108946895.9	232028470.6	168236816.7	138776077.3	2.12973916	1.544209363	1.273795606	1.9953533	ENSMUSG00000052684.5	JUN	3.72861127	0.00793651	34	-0.307312253	0.153581266
LPC(22:6)	0.89_612.33280752173	M+HCOO	108946895.9	232028470.6	168236816.7	138776077.3	2.12973916	1.544209363	1.273795606	2.3923199	ENSMUSG00000052684.5	JUN	3.72861127	0.00793651	34	-0.307312253	0.153581266
LPC(22:6)	0.89_612.33280752173	M+HCOO	108946895.9	232028470.6	168236816.7	138776077.3	2.12973916	1.544209363	1.273795606	1.9953533	ENSMUSG00000030265.15	RAS(KRAS)	5.06971122	0.00793651	34	0.438735178	0.037457123
LPC(22:6)	0.89_612.33280752173	M+HCOO	108946895.9	232028470.6	168236816.7	138776077.3	2.12973916	1.544209363	1.273795606	2.3923199	ENSMUSG00000030265.15	RAS(KRAS)	5.06971122	0.00793651	34	0.438735178	0.037457123
LPC(22:6)	0.89_612.33280752173	M+HCOO	108946895.9	232028470.6	168236816.7	138776077.3	2.12973916	1.544209363	1.273795606	1.9953533	ENSMUSG00000018932.10	MKK3(MAP2K3)	0.52906915	0.01587302	34	-0.311264822	0.148153613
LPC(22:6)	0.89_612.33280752173	M+HCOO	108946895.9	232028470.6	168236816.7	138776077.3	2.12973916	1.544209363	1.273795606	2.3923199	ENSMUSG00000018932.10	MKK3(MAP2K3)	0.52906915	0.01587302	34	-0.311264822	0.148153613
LPC(22:6)	0.89_612.33280752173	M+HCOO	108946895.9	232028470.6	168236816.7	138776077.3	2.12973916	1.544209363	1.273795606	2.3923199	ENSMUSG00000020623.12	MKK6(MAP2K6)	0.25553044	0.00793651	34	0.605731225	0.002669002
LPC(22:6)	3.07_580.43396160869	M+HCOO	108946895.9	232028470.6	168236816.7	138776077.3	2.12973916	1.544209363	1.273795606	1.206095	ENSMUSG00000020623.12	MKK6(MAP2K6)	0.25553044	0.00793651	34	0.321412173	0.134786958
LPC(22:6)	0.89_612.33280752173	M+HCOO	108946895.9	232028470.6	168236816.7	138776077.3	2.12973916	1.544209363	1.273795606	1.9953533	ENSMUSG00000002948.20	MKK7(MAP2K7)	0.29104921	0.01587302	34	-0.468102434	0.024281827
LPC(22:6)	0.89_612.33280752173	M+HCOO	108946895.9	232028470.6	168236816.7	138776077.3	2.12973916	1.544209363	1.273795606	2.3923199	ENSMUSG00000002948.20	MKK7(MAP2K7)	0.29104921	0.01587302	34	-0.468102434	0.024281827
LPC(22:6)	1.14_572.37172873913	M+HCOO	108946895.9	232028470.6	168236816.7	138776077.3	2.12973916	1.544209363	1.273795606	1.9186668	ENSMUSG00000000440.13	PPARG	2.92849265	0.05555556	34	-0.541501976	0.008510834
LPC(22:6)	0.89_612.33280752173	M+HCOO	108946895.9	232028470.6	168236816.7	138776077.3	2.12973916	1.544209363	1.273795606	1.9953533	ENSMUSG00000000440.13	PPARG	2.92849265	0.05555556	34	0.605731225	0.002669002
LPC(22:6)	1.14_572.37172873913	M+HCOO	108946895.9	232028470.6	168236816.7	138776077.3	2.12973916	1.544209363	1.273795606	1.9186668	ENSMUSG00000040435.13	GADD34(PPP1R15A)	0.50828146	0.05555556	34	0.590909091	0.003557153
LPC(22:6)	1.14_572.37172873913	M+HCOO	108946895.9	232028470.6	168236816.7	138776077.3	2.12973916	1.544209363	1.273795606	1.9186668	ENSMUSG00000040435.13	GADD34(PPP1R15A)	0.50828146	0.05555556	34	-0.335968379	0.117330976
LPC(22:6)	0.89_612.33280752173	M+HCOO	108946895.9	232028470.6	168236816.7	138776077.3	2.12973916	1.544209363	1.273795606	1.9953533	ENSMUSG00000031887.14	TRADD	0.47964951	0.05555556	34	-0.314229249	0.144174687
LPC(22:6)	0.89_612.33280752173	M+HCOO	108946895.9	232028470.6	168236816.7	138776077.3	2.12973916	1.544209363	1.273795606	2.3923199	ENSMUSG00000031887.14	TRADD	0.47964951	0.05555556	34	-0.314229249	0.144174687
LPC(22:6)	1.14_572.37172873913	M+HCOO	108946895.9	232028470.6	168236816.7	138776077.3	2.12973916	1.544209363	1.273795606	1.9186668	ENSMUSG00000026942.14	TRAF2	6.47524644	6.03174603	34	0.450592885	0.032185191
LPC(22:6)	1.14_572.37172873913	M+HCOO	108946895.9	232028470.6	168236816.7	138776077.3	2.12973916	1.544209363	1.273795606	1.9186668	ENSMUSG00000026942.14	TRAF2	7.47524644	7.03174603	34	-0.509881423	0.014017036
LPC(22:6)	1.14_572.37172873913	M+HCOO	108946895.9	232028470.6	168236816.7	138776077.3	2.12973916	1.544209363	1.273795606	1.9186668	ENSMUSG00000020484.20	XBP1	0.71865981	0.15079365	34	0.468379447	0.025413081
LPC(22:6)	1.14_572.37172873913	M+HCOO	108946895.9	232028470.6	168236816.7	138776077.3	2.12973916	1.544209363	1.273795606	1.9186668	ENSMUSG00000020484.20	XBP1	0.71865981	0.15079365	34	-0.461462451	0.027893837
LPC(22:6)	3.07_580.43396160869	M+HCOO	108946895.9	232028470.6	168236816.7	138776077.3	2.12973916	1.544209363	1.273795606	1.206095	ENSMUSG00000042406.9	ATF4	0.60874006	0.0952381	34	-0.556324111	0.006634679

AC vs CC

Lipid											Gene				Correlation		
Lipid group	Lipid ID	Adduct	Lipid aundance_CC	Lipid aundance_AA	Lipid aundance_AC	Lipid aundance_CA	ratio_AA VS CC	ratio_AC VS CC	ratio_CA VS CC	VIP	Gene ID	Gene name	Gene fold change	p value_gene fold change	Lipid-gene interactions	Spearman's rank correlation coefficiency	p value_correlation
LPC(14:0)	0.91_468.309003	M+H	6812061.999	10512651.83	14233335.7	8901674.605	1.543240774	2.089431321	1.306751848	1.6478496	ENSMUSG00000026663.7	ATF6	0.98962466	0.53679654	12	0.436758893	0.038399364
LPC(14:0)	0.91_468.309003	M+H	6812061.999	10512651.83	14233335.7	8901674.605	1.543240774	2.089431321	1.306751848	1.6478496	ENSMUSG00000021116.11	EIF2α(EIF2S1)	1.12106248	0.66233766	12	-0.562391912	0.005216828
LPC(14:0)	0.91_468.309003	M+H	6812061.999	10512651.83	14233335.7	8901674.605	1.543240774	2.089431321	1.306751848	1.6478496	ENSMUSG00000031077.8	FADD	0.36416921	0.03534433	12	0.320158103	0.136449952
LPC(14:0)	0.91_468.309003	M+H	6812061.999	10512651.83	14233335.7	8901674.605	1.543240774	2.089431321	1.306751848	1.6478496	ENSMUSG00000020048.14	HSP90B1	1.35389366	0.17748918	12	0.312252964	0.146818602
LPC(14:0)	0.91_468.309003	M+H	6812061.999	10512651.83	14233335.7	8901674.605	1.543240774	2.089431321	1.306751848	1.6478496	ENSMUSG00000004221.17	IKBKG	0.87707926	0.93073593	12	-0.377470356	0.076624247
LPC(14:0)	0.91_468.309003	M+H	6812061.999	10512651.83	14233335.7	8901674.605	1.543240774	2.089431321	1.306751848	1.6478496	ENSMUSG00000030265.15	RAS(KRAS)	1.14572018	1	12	-0.360671937	0.091536597
LPC(14:0)	0.91_468.309003	M+H	6812061.999	10512651.83	14233335.7	8901674.605	1.543240774	2.089431321	1.306751848	1.6478496	ENSMUSG00000020623.12	MKK6(MAP2K6)	0.73875525	0.66233766	12	0.307388198	0.153640865
LPC(14:0)	0.91_468.309003	M+H	6812061.999	10512651.83	14233335.7	8901674.605	1.543240774	2.089431321	1.306751848	1.6478496	ENSMUSG00000020941.8	NIK(MAP3K14)	0.82056162	0.93073593	12	-0.353138951	0.098350992
LPC(14:0)	0.91_468.309003	M+H	6812061.999	10512651.83	14233335.7	8901674.605	1.543240774	2.089431321	1.306751848	1.6478496	ENSMUSG00000071369.12	ASK1(MAP3K5)	3.13947843	0.66233766	12	0.442302954	0.034573597
LPC(14:0)	0.91_468.309003	M+H	6812061.999	10512651.83	14233335.7	8901674.605	1.543240774	2.089431321	1.306751848	1.6478496	ENSMUSG00000000440.13	PPARG	0.86292096	0.7837113	12	0.382411067	0.072615126
LPC(14:0)	0.91_468.309003	M+H	6812061.999	10512651.83	14233335.7	8901674.605	1.543240774	2.089431321	1.306751848	1.6478496	ENSMUSG00000024401.15	TNF	0	0.01502429	12	-0.39229249	0.065084096
LPC(14:0)	0.91_468.309003	M+H	6812061.999	10512651.83	14233335.7	8901674.605	1.543240774	2.089431321	1.306751848	1.6478496	ENSMUSG00000026942.14	TRAF2	0.43197736	0.03030303	12	0.37944664	0.075000674
LPC(18:2)	0.95_564.331421	M+HCOO	123530878	206174058	201817305	156682839	1.66900828	1.63373974	1.26836984	1.4608182	ENSMUSG00000004056.16	AKT2	1.53101061	0.32900433	11	0.333003953	0.1207552
LPC(18:2)	0.95_564.331421	M+HCOO	123530878	206174058	201817305	156682839	1.66900828	1.63373974	1.26836984	1.4608182	ENSMUSG00000025076.13	CASP7	1.42472646	1	11	0.314229249	0.144174687
LPC(18:2)	0.95_564.331421	M+HCOO	123530878	206174058	201817305	156682839	1.66900828	1.63373974	1.26836984	1.4608182	ENSMUSG00000022136.9	HSP40(DNAJC3)	1.45102465	0.08225108	11	0.331027668	0.12307867
LPC(18:2)	0.95_564.331421	M+HCOO	123530878	206174058	201817305	156682839	1.66900828	1.63373974	1.26836984	1.4608182	ENSMUSG00000020623.12	MKK6(MAP2K6)	0.73875525	0.66233766	11	-0.415019763	0.050065877
LPC(18:2)	0.95_564.331421	M+HCOO	123530878	206174058	201817305	156682839	1.66900828	1.63373974	1.26836984	1.4608182	ENSMUSG00000002948.20	MKK7(MAP2K7)	0.79771013	0.42857143	11	0.31027668	0.149497361
LPC(18:2)	0.95_564.331421	M+HCOO	123530878	206174058	201817305	156682839	1.66900828	1.63373974	1.26836984	1.4608182	ENSMUSG00000053436.16	p38α(MAPK14)	1.72836441	0.32900433	11	0.492581843	0.016944553
LPC(18:2)	0.95_564.331421	M+HCOO	123530878	206174058	201817305	156682839	1.66900828	1.63373974	1.26836984	1.4608182	ENSMUSG00000021936.15	JNK1(MAPK8)	2.27652328	0.7837113	11	0.33201581	0.12191285
LPC(18:2)	0.95_564.331421	M+HCOO	123530878	206174058	201817305	156682839	1.66900828	1.63373974	1.26836984	1.4608182	ENSMUSG00000020366.19	JNK2(MAPK9)	1.10747553	1	11	0.305335968	0.156347999
LPC(18:2)	0.95_564.331421	M+HCOO	123530878	206174058	201817305	156682839	1.66900828	1.63373974	1.26836984	1.4608182	ENSMUSG00000028163.18	NFKB1	1.03768403	0.79220779	11	0.392834862	0.063702653
LPC(18:2)	0.95_564.331421	M+HCOO	123530878	206174058	201817305	156682839	1.66900828	1.63373974	1.26836984	1.4608182	ENSMUSG00000028698.14	PI3K-C2γ(PIK3R7)	1.44103352	0.42857143	11	-0.527667984	0.010642279
LPC(18:2)	0.95_564.331421	M+HCOO	123530878	206174058	201817305	156682839	1.66900828	1.63373974	1.26836984	1.4608182	ENSMUSG00000030341.18	TNFRSF1A	0.66168633	0.32900433	11	0.342885375	0.109621579
LPC(18:3)	0.99_562.31581552173	M+HCOO	3149752.657	5984448.604	5419680.917	4629188.43	1.899974143	1.720668734	1.469699032	1.6018649	ENSMUSG00000022136.9	HSP40(DNAJC3)	1.45102465	0.08225108	9	0.314229249	0.144174687
LPC(18:3)	0.99_562.31581552173	M+HCOO	3149752.657	5984448.604	5419680.917	4629188.43	1.899974143	1.720668734	1.469699032	1.6018649	ENSMUSG00000031077.8	FADD	0.36416921	0.03534433	9	0.327075099	0.127824212
LPC(18:3)	0.99_562.31581552173	M+HCOO	3149752.657	5984448.604	5419680.917	4629188.43	1.899974143	1.720668734	1.469699032	1.6018649	ENSMUSG00000004221.17	IKBKG	0.87707926	0.93073593	9	-0.343873518	0.108551852
LPC(18:3)	0.99_562.31581552173	M+HCOO	3149752.657	5984448.604	5419680.917	4629188.43	1.899974143	1.720668734	1.469699032	1.6018649	ENSMUSG00000030265.15	RAS(KRAS)	1.14572018	1	9	0.464047457	0.02571218
LPC(18:3)	0.99_562.31581552173	M+HCOO	3149752.657	5984448.604	5419680.917	4629188.43	1.899974143	1.720668734	1.469699032	1.6018649	ENSMUSG00000020941.8	NIK(MAP3K14)	0.82056162	0.93073593	9	0.357263201	0.094212876
LPC(18:3)	0.99_562.31581552173	M+HCOO	3149752.657	5984448.604	5419680.917	4629188.43	1.899974143	1.720668734	1.469699032	1.6018649	ENSMUSG00000071369.12	ASK1(MAP3K5)	3.13947843	0.66233766	9	-0.386363636	0.06952597
LPC(18:3)	0.99_562.31581552173	M+HCOO	3149752.657	5984448.604	5419680.917	4629188.43	1.899974143	1.720668734	1.469699032	1.6018649	ENSMUSG00000028698.14	PI3K-C2γ(PIK3R7)	1.44103352	0.42857143	9	0.350790514	0.101280808
LPC(18:3)	0.99_562.31581552173	M+HCOO	3149752.657	5984448.604	5419680.917	4629188.43	1.899974143	1.720668734	1.469699032	1.6018649	ENSMUSG00000000441.18	RAF1	0.82434388	0.79220779	9	-0.595849802	0.003236985
LPC(18:3)	0.99_562.31581552173	M+HCOO	3149752.657	5984448.604	5419680.917	4629188.43	1.899974143	1.720668734	1.469699032	1.6018649	ENSMUSG00000020484.20	XBP1	0.94214103	0.93073593	9	-0.487768732	0.018222775
LPC(20:2)	1.23_548.37257786956	M+H	7025246.705	12492375.77	13536831.19	8649471.031	1.778211683	1.92688339	1.23119819	1.7783984	ENSMUSG00000022136.9	HSP40(DNAJC3)	1.45102465	0.08225108	16	0.427865613	0.042876506
LPC(20:2)	1.23_548.37257786956	M+H	7025246.705	12492375.77	13536831.19	8649471.031	1.778211683	1.92688339	1.23119819	1.7783984	ENSMUSG00000021116.11	EIF2α(EIF2S1)	1.12106248	0.66233766	16	-0.568322231	0.004664533
LPC(20:2)	1.23_548.37257786956	M+H	7025246.705	12492375.77	13536831.19	8649471.031	1.778211683	1.92688339	1.23119819	1.7783984	ENSMUSG00000031077.8	FADD	0.36416921	0.03534433	16	0.382411067	0.072615126
LPC(20:2)	1.23_548.37257786956	M+H	7025246.705	12492375.77	13536831.19	8649471.031	1.778211683	1.92688339	1.23119819	1.7783984	ENSMUSG00000020048.14	HSP90B1	1.35389366	0.17748918	16	0.431818182	0.040838014
LPC(20:2)	1.23_548.37257786956	M+H	7025246.705	12492375.77	13536831.19	8649471.031	1.778211683	1.92688339	1.23119819	1.7783984	ENSMUSG00000030265.15	RAS(KRAS)	1.14572018	1	16	0.389328063	0.067276608
LPC(20:2)	1.23_548.37257786956	M+H	7025246.705	12492375.77	13536831.19	8649471.031	1.778211683	1.92688339	1.23119819	1.7783984	ENSMUSG00000004936.9	MEK1(MAP2K1)	1.18670465	0.66233766	16	-0.327075099	0.127824212
LPC(20:2)	1.23_548.37257786956	M+H	7025246.705	12492375.77	13536831.19	8649471.031	1.778211683	1.92688339	1.23119819	1.7783984	ENSMUSG00000020623.12	MKK6(MAP2K6)	0.73875525	0.66233766	16	0.492094862	0.018230788
LPC(20:2)	1.23_548.37257786956	M+H	7025246.705	12492375.77	13536831.19	8649471.031	1.778211683	1.92688339	1.23119819	1.7783984	ENSMUSG00000002948.20	MKK7(MAP2K7)	0.79771013	0.42857143	16	0.330971104	0.122924292

LPC(20:2)	1.23_548.37257786956	M+H	7025246.705	12492375.77	13536831.19	8649471.031	1.778211683	1.92688339	1.23119819	1.7783984	ENSMUSG00000053436.16	p38α(MAPK14)	1.72836441	0.32900433	16	-0.302371542	0.160564772
LPC(20:2)	1.23_548.37257786956	M+H	7025246.705	12492375.77	13536831.19	8649471.031	1.778211683	1.92688339	1.23119819	1.7783984	ENSMUSG00000020366.19	JNK2(MAPK9)	1.10747553	1	16	0.308300395	0.152211162
LPC(20:2)	1.23_548.37257786956	M+H	7025246.705	12492375.77	13536831.19	8649471.031	1.778211683	1.92688339	1.23119819	1.7783984	ENSMUSG00000028698.14	PI3K-C2γ(PIK3R7)	1.44103352	0.42857143	16	-0.377470356	0.076624247
LPC(20:2)	1.23_548.37257786956	M+H	7025246.705	12492375.77	13536831.19	8649471.031	1.778211683	1.92688339	1.23119819	1.7783984	ENSMUSG00000000440.13	PPARG	0.86292096	0.7837113	16	-0.389741674	0.066007645
LPC(20:2)	1.23_548.37257786956	M+H	7025246.705	12492375.77	13536831.19	8649471.031	1.778211683	1.92688339	1.23119819	1.7783984	ENSMUSG00000000441.18	RAF1	0.82434388	0.79220779	16	-0.380434783	0.074198883
LPC(20:2)	1.23_548.37257786956	M+H	7025246.705	12492375.77	13536831.19	8649471.031	1.778211683	1.92688339	1.23119819	1.7783984	ENSMUSG00000031887.14	TRADD	1.31630323	1	16	-0.396245059	0.062247604
LPC(20:2)	1.23_548.37257786956	M+H	7025246.705	12492375.77	13536831.19	8649471.031	1.778211683	1.92688339	1.23119819	1.7783984	ENSMUSG00000026942.14	TRAF2	0.43197736	0.03030303	16	0.316205534	0.141565411
LPC(20:2)	1.23_548.37257786956	M+H	7025246.705	12492375.77	13536831.19	8649471.031	1.778211683	1.92688339	1.23119819	1.7783984	ENSMUSG00000020484.20	XBP1	0.94214103	0.93073593	16	-0.513833992	0.013199547
LPC(20:4)	0.93_588.33103530434	M+HCOO	150822398.6	267574750.1	230639562.1	219933215.7	1.774104858	1.52921293	1.458226482	1.3257078	ENSMUSG00000026029.15	CASP8	0.9900005	0.79220779	14	0.300395257	0.163420675
LPC(20:4)	0.93_588.33103530434	M+HCOO	150822398.6	267574750.1	230639562.1	219933215.7	1.774104858	1.52921293	1.458226482	1.3257078	ENSMUSG00000021116.11	EIF2α(EIF2S1)	1.12106248	0.66233766	14	0.298986913	0.165786306
LPC(20:4)	0.93_588.33103530434	M+HCOO	150822398.6	267574750.1	230639562.1	219933215.7	1.774104858	1.52921293	1.458226482	1.3257078	ENSMUSG00000031077.8	FADD	0.36416921	0.03534433	14	-0.376482213	0.077446096
LPC(20:4)	0.93_588.33103530434	M+HCOO	150822398.6	267574750.1	230639562.1	219933215.7	1.774104858	1.52921293	1.458226482	1.3257078	ENSMUSG00000004221.17	IKBKG	0.87707926	0.93073593	14	0.364624506	0.087844503
LPC(20:4)	0.93_588.33103530434	M+HCOO	150822398.6	267574750.1	230639562.1	219933215.7	1.774104858	1.52921293	1.458226482	1.3257078	ENSMUSG00000030265.15	RAS(KRAS)	1.14572018	1	14	0.424282272	0.043613121
LPC(20:4)	0.93_588.33103530434	M+HCOO	150822398.6	267574750.1	230639562.1	219933215.7	1.774104858	1.52921293	1.458226482	1.3257078	ENSMUSG00000020623.12	MKK6(MAP2K6)	0.73875525	0.66233766	14	0.330615281	0.123351845
LPC(20:4)	0.93_588.33103530434	M+HCOO	150822398.6	267574750.1	230639562.1	219933215.7	1.774104858	1.52921293	1.458226482	1.3257078	ENSMUSG00000020941.8	NIK(MAP3K14)	0.82056162	0.93073593	14	-0.385375494	0.070288548
LPC(20:4)	0.93_588.33103530434	M+HCOO	150822398.6	267574750.1	230639562.1	219933215.7	1.774104858	1.52921293	1.458226482	1.3257078	ENSMUSG00000071369.12	ASK1(MAP3K5)	3.13947843	0.66233766	14	-0.640316206	0.001292554
LPC(20:4)	0.93_588.33103530434	M+HCOO	150822398.6	267574750.1	230639562.1	219933215.7	1.774104858	1.52921293	1.458226482	1.3257078	ENSMUSG00000053436.16	p38α(MAPK14)	1.72836441	0.32900433	14	-0.301383399	0.161988236
LPC(20:4)	0.93_588.33103530434	M+HCOO	150822398.6	267574750.1	230639562.1	219933215.7	1.774104858	1.52921293	1.458226482	1.3257078	ENSMUSG00000028698.14	PI3K-C2γ(PIK3R7)	1.44103352	0.42857143	14	-0.433794466	0.039848155
LPC(20:4)	0.93_588.33103530434	M+HCOO	150822398.6	267574750.1	230639562.1	219933215.7	1.774104858	1.52921293	1.458226482	1.3257078	ENSMUSG00000000440.13	PPARG	0.86292096	0.7837113	14	0.338522373	0.114101774
LPC(20:4)	0.93_588.33103530434	M+HCOO	150822398.6	267574750.1	230639562.1	219933215.7	1.774104858	1.52921293	1.458226482	1.3257078	ENSMUSG00000040435.13	GADD34(PPP1R15A)	0.72194352	0.24675325	14	0.311264822	0.148153613
LPC(20:4)	0.93_588.33103530434	M+HCOO	150822398.6	267574750.1	230639562.1	219933215.7	1.774104858	1.52921293	1.458226482	1.3257078	ENSMUSG00000026942.14	TRAF2	0.43197736	0.03030303	14	0.445652174	0.034304023
LPC(20:4)	0.93_588.33103530434	M+HCOO	150822398.6	267574750.1	230639562.1	219933215.7	1.774104858	1.52921293	1.458226482	1.3257078	ENSMUSG00000020484.20	XBP1	0.94214103	0.93073593	14	0.473320158	0.023753305
LPC(20:4)	0.94_544.34120230434	M+H	1025081106	1946614008	1742498230	1503639564	1.898985355	1.699863767	1.46684936	1.5621311	ENSMUSG00000022136.9	HSP40(DNAJC3)	1.45102465	0.08225108	15	-0.37055336	0.082520168
LPC(20:4)	0.94_544.34120230434	M+H	1025081106	1946614008	1742498230	1503639564	1.898985355	1.699863767	1.46684936	1.5621311	ENSMUSG00000031077.8	FADD	0.36416921	0.03534433	15	0.311264822	0.148153613
LPC(20:4)	0.94_544.34120230434	M+H	1025081106	1946614008	1742498230	1503639564	1.898985355	1.699863767	1.46684936	1.5621311	ENSMUSG00000004221.17	IKBKG	0.87707926	0.93073593	15	-0.429841897	0.041847377
LPC(20:4)	0.94_544.34120230434	M+H	1025081106	1946614008	1742498230	1503639564	1.898985355	1.699863767	1.46684936	1.5621311	ENSMUSG00000030265.15	RAS(KRAS)	1.14572018	1	15	-0.483568374	0.019401112
LPC(20:4)	0.94_544.34120230434	M+H	1025081106	1946614008	1742498230	1503639564	1.898985355	1.699863767	1.46684936	1.5621311	ENSMUSG00000020623.12	MKK6(MAP2K6)	0.73875525	0.66233766	15	0.317193676	0.140273699
LPC(20:4)	0.94_544.34120230434	M+H	1025081106	1946614008	1742498230	1503639564	1.898985355	1.699863767	1.46684936	1.5621311	ENSMUSG00000020941.8	NIK(MAP3K14)	0.82056162	0.93073593	15	0.392834862	0.063702653
LPC(20:4)	0.94_544.34120230434	M+H	1025081106	1946614008	1742498230	1503639564	1.898985355	1.699863767	1.46684936	1.5621311	ENSMUSG00000053436.16	p38α(MAPK14)	1.72836441	0.32900433	15	-0.385375494	0.070288548
LPC(20:4)	0.94_544.34120230434	M+H	1025081106	1946614008	1742498230	1503639564	1.898985355	1.699863767	1.46684936	1.5621311	ENSMUSG00000028698.14	PI3K-C2γ(PIK3R7)	1.44103352	0.42857143	15	0.311264822	0.148153613
LPC(20:4)	0.94_544.34120230434	M+H	1025081106	1946614008	1742498230	1503639564	1.898985355	1.699863767	1.46684936	1.5621311	ENSMUSG00000000440.13	PPARG	0.86292096	0.7837113	15	0.368577075	0.084266788
LPC(20:4)	0.94_544.34120230434	M+H	1025081106	1946614008	1742498230	1503639564	1.898985355	1.699863767	1.46684936	1.5621311	ENSMUSG00000040435.13	GADD34(PPP1R15A)	0.72194352	0.24675325	15	0.374104286	0.078647267
LPC(20:4)	0.94_544.34120230434	M+H	1025081106	1946614008	1742498230	1503639564	1.898985355	1.699863767	1.46684936	1.5621311	ENSMUSG00000000441.18	RAF1	0.82434388	0.79220779	15	-0.336956522	0.116205712
LPC(22:0)	1.24_592.36229652173	M+H	1939988.712	2498346.356	2420156.701	2389134.987	1.287814893	1.247510713	1.231520046	1.1606002	ENSMUSG00000027398.14	IL1β(IL1B)	0.1420621	0.00865801	15	0.47173913	0.047035573
LPC(22:0)	1.24_592.36229652173	M+H	1939988.712	2498346.356	2420156.701	2389134.987	1.287814893	1.247510713	1.231520046	1.1606002	ENSMUSG00000020048.14	HSP90B1	1.35389366	0.17748918	15	0.551778656	0.030272723
LPC(22:0)	1.24_592.36229652173	M+H	1939988.712	2498346.356	2420156.701	2389134.987	1.287814893	1.247510713	1.231520046	1.1606002	ENSMUSG00000026864.14	GRP78	0.76470587	0.66233766	15	0.617391304	0.017522408
LPC(22:0)	1.24_592.36229652173	M+H	1939988.712	2498346.356	2420156.701	2389134.987	1.287814893	1.247510713	1.231520046	1.1606002	ENSMUSG00000029380.12	CXCL1	0.89346113	0.42857143	15	-0.638538159	0.00104147
LPC(22:6)	0.89_612.33280752173	M+HCOO	108946895.9	232028470.6	168236816.7	138776077.3	2.12973916	1.544209363	1.273795606	1.275505	ENSMUSG00000042406.9	ATF4	0.72063553	0.17748918	17	0.540513834	0.00865021
LPC(22:6)	0.89_612.33280752173	M+HCOO	108946895.9	232028470.6	168236816.7	138776077.3	2.12973916	1.544209363	1.273795606	1.275505	ENSMUSG00000026029.15	CASP8	0.9900005	0.79220779	17	-0.314229249	0.144174687
LPC(22:6)	0.89_612.33280752173	M+HCOO	108946895.9	232028470.6	168236816.7	138776077.3	2.12973916	1.544209363	1.273795606	1.275505	ENSMUSG00000022136.9	HSP40(DNAJC3)	1.45102465	0.08225108	17	0.476284585	0.022800557
LPC(22:6)	0.89_612.33280752173	M+HCOO	108946895.9	232028470.6	168236816.7	138776077.3	2.12973916	1.544209363	1.273795606	1.275505	ENSMUSG00000021116.11	EIF2α(EIF2S1)	1.12106248	0.66233766	17	-0.508524849	0.013220356
LPC(22:6)	0.89_612.33280752173	M+HCOO	108946895.9	232028470.6	168236816.7	138776077.3	2.12973916	1.544209363	1.273795606	1.275505	ENSMUSG00000031077.8	FADD	0.36416921	0.03534433	17	0.330039526	0.124252687
LPC(22:6)	0.89_612.33280752173	M+HCOO	108946895.9	232028470.6	168236816.7	138776077.3	2.12973916	1.544209363	1.273795606	1.275505	ENSMUSG00000020048.14	HSP90B1	1.35389366	0.17748918	17	0.513833992	0.013199547
LPC(22:6)	0.89_612.33280752173	M+HCOO	108946895.9	232028470.6	168236816.7	138776077.3	2.12973916	1.544209363	1.273795606	1.275505	ENSMUSG00000004221.17	IKBKG	0.87707926	0.93073593	17	-0.460474308	0.028263706
LPC(22:6)	0.89_612.33280752173	M+HCOO	108946895.9	232028470.6	168236816.7	138776077.3	2.12973916	1.544209363	1.273795606	1.275505	ENSMUSG00000052684.5	JUN	0.64618646	0.32900433	17	-0.468102434	0.024281827
LPC(22:6)	0.89_612.33280752173	M+HCOO	108946895.9	232028470.6	168236816.7	138776077.3	2.12973916	1.544209363	1.273795606	1.275505	ENSMUSG00000030265.15	RAS(KRAS)	1.14572018	1	17	0.481838414	0.019903993
LPC(22:6)	0.89_612.33280752173	M+HCOO	108946895.9	232028470.6	168236816.7	138776077.3	2.12973916	1.544209363	1.273795606	1.275505	ENSMUSG00000018932.10	MKK3(MAP2K3)	1.03240509	0.93073593	17	-0.419960474	0.047195951
LPC(22:6)	0.89_612.33280752173	M+HCOO	108946895.9	232028470.6	168236816.7	138776077.3	2.12973916	1.544209363	1.273795606	1.275505	ENSMUSG00000020623.12	MKK6(MAP2K6)	0.73875525	0.66233766	17	-0.342885375	0.109621579
LPC(22:6)	0.89_612.33280752173	M+HCOO	108946895.9	232028470.6	168236816.7	138776077.3	2.12973916	1.544209363	1.273795606	1.275505	ENSMUSG00000002948.20	MKK7(MAP2K7)	0.79771013	0.42857143	17	0.468379447	0.025413081
LPC(22:6)	0.89_612.33280752173	M+HCOO	108946895.9	232028470.6	168236816.7	138776077.3	2.12973916	1.544209363	1.273795606	1.275505	ENSMUSG00000000440.13	PPARG	0.86292096	0.7837113	17	-0.307312253	0.153581266
LPC(22:6)	0.89_612.33280752173	M+HCOO	108946895.9	232028470.6	168236816.7	138776077.3	2.12973916	1.544209363	1.273795606	1.275505	ENSMUSG00000040435.13	GADD34(PPP1R15A)	0.72194352	0.24675325	17	0.438735178	0.037457123
LPC(22:6)	0.89_612.33280752173	M+HCOO	108946895.9	232028470.6	168236816.7	138776077.3	2.12973916	1.544209363	1.273795606	1.275505	ENSMUSG00000031887.14	TRADD	1.31630323	1	17	-0.483201581	0.020698212
LPC(22:6)	0.89_612.33280752173	M+HCOO	108946895.9	232028470.6	168236816.7	138776077.3	2.12973916	1.544209363	1.273795606	1.275505	ENSMUSG00000026942.14	TRAF2	0.43197736	0.03030303	17	0.342885375	0.109621579
LPC(22:6)	0.89_612.33280752173	M+HCOO	108946895.9	232028470.6	168236816.7	138776077.3	2.12973916	1.544209363	1.273795606	1.275505	ENSMUSG00000020484.20	XBP1	0.94214103	0.93073593	17	0.605731225	0.002669002

CA vs CC

Lipid											Gene				Correlation		
Lipid group	Lipid ID	Adduct	Lipid aundance_CC	Lipid aundance_AA	Lipid aundance_AC	Lipid aundance_CA	ratio_AA VS CC	ratio_AC VS CC	ratio_CA VS CC	VIP	Gene ID	Gene name	Gene fold change	p value_gene fold change	Lipid-gene interactions	Spearman's rank correlation coefficiency	p value_correlation
LPC(17:0)	1.33_554.34716630434	M+HCOO	4955061.455	9100598.091	5480528.492	8558826.17	1.836626684	1.106046523	1.72728961	1.6432448	ENSMUSG00000004221.17	IKBKG	1.69294194	0.28571429	5	0.309364971	0.150876708
LPC(17:0)	1.33_554.34716630434	M+HCOO	4955061.455	9100598.091	5480528.492	8558826.17	1.836626684	1.106046523	1.72728961	1.6432448	ENSMUSG00000030265.15	RAS(KRAS)	1.94591504	0.28571429	5	0.400197628	0.059508395
LPC(17:0)	1.33_554.34716630434	M+HCOO	4955061.455	9100598.091	5480528.492	8558826.17	1.836626684	1.106046523	1.72728961	1.6432448	ENSMUSG00000018932.10	MEK1(MAP2K3)	0.34161508	0.06349206	5	-0.327075099	0.127824212
LPC(17:0)	1.33_554.34716630434	M+HCOO	4955061.455	9100598.091	5480528.492	8558826.17	1.836626684	1.106046523	1.72728961	1.6432448	ENSMUSG00000000440.13	PPARG	1.62956735	0.73015873	5	-0.333992095	0.119605691
LPC(17:0)	1.33_554.34716630434	M+HCOO	4955061.455	9100598.091	5480528.492	8558826.17	1.836626684	1.106046523	1.72728961	1.6432448	ENSMUSG00000020484.20	XBP1	0.94543785	0.90476191	5	0.432806324	0.040340663
LPC(20:4)	0.93_588.33103530434	M+HCOO	150822398.6	267574750.1	230639562.1	219933215.7	1.774104858	1.52921293	1.458226482	1.1368411	ENSMUSG00000026029.15	CASP8	0.99020968	0.55555556	14	0.300395257	0.163420675
LPC(20:4)	0.93_588.33103530434	M+HCOO	150822398.6	267574750.1	230639562.1	219933215.7	1.774104858	1.52921293	1.458226482	1.1368411	ENSMUSG00000021116.11	EIF2α(EIF2S1)	0.8713626	0.73015873	14	0.298986913	0.165786306
LPC(20:4)	0.93_588.33103530434	M+HCOO	150822398.6	267574750.1	230639562.1	219933215.7	1.774104858	1.52921293	1.458226482	1.1368411	ENSMUSG00000031077.8	FADD	0.71260063	0.28571429	14	-0.376482213	0.077446096
LPC(20:4)	0.93_588.33103530434	M+HCOO	150822398.6	267574750.1	230639562.1	219933215.7	1.774104858	1.52921293	1.458226482	1.1368411	ENSMUSG00000004221.17	IKBKG	1.69294194	0.28571429	14	0.364624506	0.087844503
LPC(20:4)	0.93_588.33103530434	M+HCOO	150822398.6	267574750.1	230639562.1	219933215.7	1.774104858	1.52921293	1.458226482	1.1368411	ENSMUSG00000030265.15	RAS(KRAS)	1.94591504	0.28571429	14	0.424282272	0.043613121

LPC(20:4)	0.93_588.33103530434	M+HCOO	150822398.6	267574750.1	230639562.1	219933215.7	1.774104858	1.52921293	1.458226482	1.1368411	ENSMUSG00000020623.12	MKK6(MAP2K6)	1.14133231	0.90476191	14	0.330615281	0.123351845
LPC(20:4)	0.93_588.33103530434	M+HCOO	150822398.6	267574750.1	230639562.1	219933215.7	1.774104858	1.52921293	1.458226482	1.1368411	ENSMUSG00000020941.8	NIK(MAP3K14)	0.51056532	0.28571429	14	-0.385375494	0.070288548
LPC(20:4)	0.93_588.33103530434	M+HCOO	150822398.6	267574750.1	230639562.1	219933215.7	1.774104858	1.52921293	1.458226482	1.1368411	ENSMUSG00000071369.12	ASK1(MAP3K5)	1.00512766	1	14	-0.640316206	0.001292554
LPC(20:4)	0.93_588.33103530434	M+HCOO	150822398.6	267574750.1	230639562.1	219933215.7	1.774104858	1.52921293	1.458226482	1.1368411	ENSMUSG00000053436.16	p38α(MAPK14)	1.45981711	1	14	-0.301383399	0.161988236
LPC(20:4)	0.93_588.33103530434	M+HCOO	150822398.6	267574750.1	230639562.1	219933215.7	1.774104858	1.52921293	1.458226482	1.1368411	ENSMUSG00000028698.14	PI3K-C2γ(PIK3R7)	1.39410128	0.90476191	14	-0.433794466	0.039848155
LPC(20:4)	0.93_588.33103530434	M+HCOO	150822398.6	267574750.1	230639562.1	219933215.7	1.774104858	1.52921293	1.458226482	1.1368411	ENSMUSG00000000440.13	PPARG	1.62956735	0.73015873	14	0.338522373	0.114101774
LPC(20:4)	0.93_588.33103530434	M+HCOO	150822398.6	267574750.1	230639562.1	219933215.7	1.774104858	1.52921293	1.458226482	1.1368411	ENSMUSG00000040435.13	GADD34(PPP1R15A)	0.62692581	0.19047619	14	0.311264822	0.148153613
LPC(20:4)	0.93_588.33103530434	M+HCOO	150822398.6	267574750.1	230639562.1	219933215.7	1.774104858	1.52921293	1.458226482	1.1368411	ENSMUSG00000026942.14	TRAF2	0.80226122	0.41269841	14	0.445652174	0.034304023
LPC(20:4)	0.93_588.33103530434	M+HCOO	150822398.6	267574750.1	230639562.1	219933215.7	1.774104858	1.52921293	1.458226482	1.1368411	ENSMUSG00000020484.20	XBP1	0.94543785	0.90476191	14	0.473320158	0.023753305
LPC(22:0)	3.07_580.43396160869	M+H	1939988.712	2498346.356	2420156.701	2389134.987	1.287814893	1.247510713	1.231520046	1.0944582	ENSMUSG00000026663.7	ATF6	1.10561599	0.41269841	21	0.345935271	0.10589902
LPC(22:0)	3.07_580.43396160869	M+H	1939988.712	2498346.356	2420156.701	2389134.987	1.287814893	1.247510713	1.231520046	1.0944582	ENSMUSG00000031628.10	CASP3	1.98955257	0.28571429	21	0.301383399	0.161988236
LPC(22:0)	3.07_580.43396160869	M+H	1939988.712	2498346.356	2420156.701	2389134.987	1.287814893	1.247510713	1.231520046	1.0944582	ENSMUSG00000025076.13	CASP7	0.63988495	0.26223935	21	0.343224698	0.108846539
LPC(22:0)	3.07_580.43396160869	M+H	1939988.712	2498346.356	2420156.701	2389134.987	1.287814893	1.247510713	1.231520046	1.0944582	ENSMUSG00000028914.14	CASP9	0.93320589	1	21	-0.414562935	0.04920238
LPC(22:0)	3.07_580.43396160869	M+H	1939988.712	2498346.356	2420156.701	2389134.987	1.287814893	1.247510713	1.231520046	1.0944582	ENSMUSG00000020715.10	IRE1α(ERN1)	1.99599939	0.19047619	21	-0.491228085	0.017296473
LPC(22:0)	3.07_580.43396160869	M+H	1939988.712	2498346.356	2420156.701	2389134.987	1.287814893	1.247510713	1.231520046	1.0944582	ENSMUSG00000031077.8	FADD	0.71260063	0.28571429	21	0.357795909	0.093687955
LPC(22:0)	3.07_580.43396160869	M+H	1939988.712	2498346.356	2420156.701	2389134.987	1.287814893	1.247510713	1.231520046	1.0944582	ENSMUSG00000020048.14	HSP90B1	1.15669542	0.73015873	21	0.351778656	0.100272723
LPC(22:0)	3.07_580.43396160869	M+H	1939988.712	2498346.356	2420156.701	2389134.987	1.287814893	1.247510713	1.231520046	1.0944582	ENSMUSG00000030265.15	RAS(KRAS)	1.94591504	0.28571429	21	0.321412173	0.134786958
LPC(22:0)	3.07_580.43396160869	M+H	1939988.712	2498346.356	2420156.701	2389134.987	1.287814893	1.247510713	1.231520046	1.0944582	ENSMUSG00000018932.10	MEK1(MAP2K3)	0.34161508	0.06349206	21	0.447244886	0.032373842
LPC(22:0)	3.07_580.43396160869	M+H	1939988.712	2498346.356	2420156.701	2389134.987	1.287814893	1.247510713	1.231520046	1.0944582	ENSMUSG00000020623.12	MKK6(MAP2K6)	1.14133231	0.90476191	21	-0.360080351	0.091461514
LPC(22:0)	3.07_580.43396160869	M+H	1939988.712	2498346.356	2420156.701	2389134.987	1.287814893	1.247510713	1.231520046	1.0944582	ENSMUSG00000022610.11	JNK2(MAPK12)	0.58036397	0.19047619	21	0.433234633	0.038916222
LPC(22:0)	3.07_580.43396160869	M+H	1939988.712	2498346.356	2420156.701	2389134.987	1.287814893	1.247510713	1.231520046	1.0944582	ENSMUSG00000004864.14	JNK2(MAPK11)	0.58036397	0.19047619	21	-0.316205534	0.141565411
LPC(22:0)	3.07_580.43396160869	M+H	1939988.712	2498346.356	2420156.701	2389134.987	1.287814893	1.247510713	1.231520046	1.0944582	ENSMUSG00000053436.16	p38α(MAPK14)	1.45981711	1	21	-0.556324111	0.006634679
LPC(22:0)	3.07_580.43396160869	M+H	1939988.712	2498346.356	2420156.701	2389134.987	1.287814893	1.247510713	1.231520046	1.0944582	ENSMUSG00000021936.15	JNK1(MAPK8)	1.21662519	1	21	0.308300395	0.152211162
LPC(22:0)	3.07_580.43396160869	M+H	1939988.712	2498346.356	2420156.701	2389134.987	1.287814893	1.247510713	1.231520046	1.0944582	ENSMUSG00000040435.13	GADD34(PPP1R15A)	0.62692581	0.19047619	21	-0.342885375	0.109621579
LPC(22:0)	3.07_580.43396160869	M+H	1939988.712	2498346.356	2420156.701	2389134.987	1.287814893	1.247510713	1.231520046	1.0944582	ENSMUSG00000024401.15	TNF	0	0.04417135	21	-0.442687747	0.035628166
LPC(22:0)	3.07_580.43396160869	M+H	1939988.712	2498346.356	2420156.701	2389134.987	1.287814893	1.247510713	1.231520046	1.0944582	ENSMUSG00000026942.14	TRAF2	0.80226122	0.41269841	21	-0.335968379	0.117330976
LPC(22:0)	1.24_592.36229652173	M+H	1939988.712	2498346.356	2420156.701	2389134.987	1.287814893	1.247510713	1.231520046	1.0944582	ENSMUSG00000027398.14	IL1β(IL1B)	0.30509779	0.11111111	21	0.47173913	0.047035573
LPC(22:0)	1.24_592.36229652173	M+H	1939988.712	2498346.356	2420156.701	2389134.987	1.287814893	1.247510713	1.231520046	1.0944582	ENSMUSG00000020048.14	HSP90B1	1.15669542	0.73015873	21	0.551778656	0.030272723
LPC(22:0)	1.24_592.36229652173	M+H	1939988.712	2498346.356	2420156.701	2389134.987	1.287814893	1.247510713	1.231520046	1.0944582	ENSMUSG00000026864.14	GRP78	0.63745924	0.73015873	21	0.617391304	0.017522408
LPC(22:0)	1.24_592.36229652173	M+H	1939988.712	2498346.356	2420156.701	2389134.987	1.287814893	1.247510713	1.231520046	1.0944582	ENSMUSG00000029380.12	CXCL1	0.77017995	0.26834059	21	-0.638538159	0.00104147

Table S3. Primers used in the study

Human HMGCR Forward	TGATTGACCTTTCCAGAGCAAG
Human HMGCR Reverse	CTAAAATTGCCATTCCACGAGC
Human HMGCS1 Forward	GATGTGGGAATTGTTGCCCTT
Human HMGCS1 Reverse	ATTGTCTCTGTTCCAACTTCCAG
Human LSS Forward	CCGGATACAGAGAAGAGATTGTG
Human LSS Reverse	GTGCCCAGTCAGGAAACAG
Human CYP7B1 Forward	AAAGGTTGGCTTCCTTATCTTGG
Human CYP7B1 Reverse	GCAACTGACTGATGCTAAATGCT
Human CYP27A1 Forward	GGTGCTTTACAAGGCCAAGTA
Human CYP27A1 Reverse	TCCCGGTGCTCCTTCCATAG
Human ABCG1 Forward	ATGTAGGCAGATTGGTGGTTT
Human ABCG1 Reverse	TGCTAAGGAGCGACTGGACT
Mouse PLA2G10 Forward	TTGGCATTTGTTCTCTGCTG
Mouse PLAG10 Reverse	TTGGCATTTGTTCTCTGCTG
Human PLA2G10 Forward	CCTGGTCACACTTGCACAAC
Human PLAG10 Reverse	CCAAGACAGAGCGCTACTCC
Human GAPDH Forward	ATTCCACCCATGGCAAATTC
Human GAPDH Reverse	CGCTCCTGGAAGATGGTGAT
Mouse β-actin Forward	AAGGCCAACCGTGAAAAGAT
Mouse β-actin Reverse	GTGGTACGACCAGAGGCATAC
